# Supplementary material for: Quantifying electronic and geometric effects on the activity of platinum catalysts for water-gas shift
Source: Nat Commun. 2025 Jul 18;16:6641. doi: 10.1038/s41467-025-61895-8 (PMC12274351; doi:10.1038/s41467-025-61895-8)
Supplement: Supplementary file 1 — Supplementary information [file 41467_2025_61895_MOESM1_ESM.pdf]

Supplementary Information

Quantifying electronic and geometric effects on the activity of platinum catalysts for water-gas shift

Xiansheng Li[1,2,3,#], Xing Wang[2,4,#], Arik Beck[1,2], Mikalai Artsiusheuski[1,2], Qianyu Liu[5], Qiang Liu[2], Henrik Eliasson[3], Frank Krumeich[1], Ulrich Aschauer[4,6], Giovanni Pizzi[2,7,8], Rolf Erni[3], Jeroen A. van Bokhoven[1,2,*], Luca Artiglia[2,*]

[1]Institute for Chemical and Bioengineering, ETH Zurich, 8092 Zurich, Switzerland

[2]Paul Scherrer Institute, 5232 Villigen, Switzerland

[3]Electron Microscopy Center, Empa – Swiss Federal Laboratories for Materials Science and Technology, 8600 Dübendorf, Switzerland

[4]Department of Chemistry, Biochemistry and Pharmaceutical Sciences, University of Bern, 3012 Bern, Switzerland

[5]Institute of Informatics, University of Zurich, 8050 Zurich, Switzerland

[6]Department of Chemistry and Physics of Materials, University of Salzburg, 5020 Salzburg, Austria

[7]Theory and Simulation of Materials (THEOS), École Polytechnique Fédérale de Lausanne, 1015 Lausanne, Switzerland

[8] National Centre for Computational Design and Discovery of Novel Materials (MARVEL), École Polytechnique Fédérale de Lausanne, 1015 Lausanne, Switzerland

[#]Equally contributing authors

*Corresponding author Email: jeroen.vanbokhoven@chem.ethz.ch; luca.artiglia@psi.ch

The file includes:

Supplementary Methods

Supplementary Figures 1 to 45

Supplementary Tables 1 to 17

Supplementary References

S1. Supplementary Methods

S1.1 Peak fitting strategy of *Pt 4f* photoelectron spectroscopy

The proposed peak fitting strategy is based on the following assumptions:

1) Platinum on the catalyst surface consists of AD Pt^{2+} and Pt NPs.

2) The platinum nanoparticle consists of atoms at the bulk, terrace, edge and corner positions. Their fraction can be determined by geometric effect as a function of the NP diameter. The BE values of these sites are in line with the values in ref[1].

The *Pt 4f* spectrum is deconvoluted into two peak families, AD and NP. We provide extra Illustrations Supplementary Figure 15 to clarify how AD and NP contribute to the overall spectrum. The NP peak further consists of bulk, terrace, edge and corner components, which are all co-present in metal NPs. Fitting parameters were rigorously constrained (specifically, the position, FWHM, shape, and area ratio between components, see Supplementary Tables 3 and 5) throughout the whole series of collected spectra. Although multiple components were used in the deconvolution, they were not freely fitted; rather, any change in one component systematically influenced the others. In this way, the overall NP signal behaves as a tightly coupled multiplet, enhancing reproducibility and minimizing the risk of overfitting. The main goal of using this framework, with its rigorous parameters and constraints, is to provide consistent and reliable interpretations of photoemission spectra.

The theoretical platinum NP model enables the extraction of the specific ratio of those NP species at the average NP sizes obtained from the STEM images in Supplementary Table 4. Therefore, the parameters can be constrained accordingly during peak fittings of the *Pt 4f*. Supplementary Figure 15 shows an example of fitting. The average size of the STEM statistics can be used as a guide to identify the correct model. While the intrinsic resolution of XPS instrument (~0.3eV) imposes limitations on resolving edge and corner peaks, our analysis does not rely on resolving these components individually. These components were treated as a single peak with a shared binding energy (72.2 eV for $4f_{7/2}$), and their relative areas were determined by geometric modeling based on the particle size distribution extracted from STEM.

We acknowledge the inherent spatial mismatch between STEM and XPS in terms of sampling volume. While STEM provides high-resolution structural information in the

nm range, thus it is limited to local regions of the sample, XPS captures a global overview over a larger area (approximately 300 μm X-ray beam spot size). This difference introduces potential uncertainty when using localized STEM images to guide the fitting of photoemission peaks. To minimize this risk, the fitting strategy has been improved to ensure the robustness. In practical situations, when AD species start to sinter into assemblies of a few atoms, it is difficult to identify and account for these smaller clusters by electron microscopy. The proportion of small clusters may be higher, which leads to a higher proportion of corners. Therefore, we added the component "corner2" to fit the potential corners from smaller clusters. It has the same BE, FWHM, G/L ratio and asymmetry as "corner1", but the area is not constrained. "Corner2" has a negligible contribution upon AD species sintering into large platinum particles, but it becomes prominent during structural evolutions, e.g. AD_1 at 250°C during ramp-up (Supplementary Figure 16(B)). The corner sites will eventually be the sum of "corner1" and "corner2". Supplementary Figures 16 and 17 give the fitting details for all the *Pt 4f* spectra in this work. All the spectra can be fitted well by means of this strategy. In this sense, global spectral fitting can also provide complementary information to microscopy, highlighting the synergistic potential of combining both techniques.

We have assessed the error associated to fittings using the Relative Root Mean Squared Error (RRMSE) in Supplementary Table 6 by comparing the relative deviation of the fitting data from the raw spectra. The spectra exhibit a fitting error below 10%, which is considered excellent for model accuracy[2, 3, 4].

The indicator is calculated as follows:

Relative Root Mean Squared Error: $$RRMSE = \frac{\sqrt{\frac{\Sigma\left(I_{raw}-I_{fit}\right)^2}{df}}}{\overline{I_{raw}}} \quad (1)$$

Where:

$\overline{I_{raw}}$ is the average of spectrum raw intensity;

I_{fit} is the fitting intensity;

df is the degrees of freedom, which is the sample size minus the number of parameters we're trying to estimate.

The fitting error of components are then calculated counting their fraction according to the propagation of uncertainty[5]. The overall error is calculated by the root of the sum of error squares from XPS measurement and peak fitting.

We also re-processed the spectra in Supplementary Figure 18 using conventional Pt 4f fitting strategies[6, 7, 8, 9] for comparison. The “traditional” method in Supplementary Figure 18 (A) that employs two components (Pt^0 at ~71.0 eV and Pt^{2+} at ~72.8 eV) was unable to adequately fit the spectra of samples characterized by small nanoparticle sizes and a high proportion of unsaturated sites. In order to address this issue, an additional component (edge & corner at 72.2 eV) was incorporated as shown in Supplementary Figure 18 (B), resulting in an improved but still imperfect fit to the raw data. Subsequently, four components were employed in Supplementary Figure 18 (C) to achieve an optimized fit to the raw data. Notably, these four peak positions correspond to different Pt sites of the nanoparticle. Consequently, it was decided to explore whether fractions of Pt sites correspond to the Pt NP models at different sizes. The fitting result in Supplementary Figure 18 (D) indicates that the average NP size can indeed guide the fitting of Pt 4f, albeit at the expense of slightly compromising the fit quality. It should be acknowledged that the determination of NP size was conducted under vacuum conditions and entails a certain degree of error. Nonetheless, the aforementioned comparison further reinforces the significance of our tailored fitting approach.

Our current fitting approach, while not without its limitations, represents a balance we have achieved between accuracy and consistency across all spectra. We acknowledge that freeing the constraints on peaks area could potentially create more accurate peak shapes for individual spectra, but it would introduce significant variability and inconsistency across the set of spectra, undermining the comparative analysis. Therefore, our fitting approach is a deliberate balance aimed at achieving the most effective fit for individual spectra while ensuring a consistent and defensible fitting strategy across all spectra.

S1.2 Representation of supported platinum NPs via the truncated octahedral model

We established theoretical platinum NP models supported on ceria with different platinum atom numbers and, thus, different diameters (Supplementary Table 4).

Under equilibrium conditions, supported nanoparticles tend to adopt the so-called Winterbottom-like shape, which minimizes the sum of the surface and interfacial energies[10, 11]. For face-centered cubic (FCC) metals like Pt, the (111) surface is typically the lowest energy in vacuum and tends to be exposed[12, 13]. For these metallic systems, the Winterbottom-like shape often gives rise to a truncated Wulff shape[14]. Based on the classic Winterbottom-like shapes of supported clusters for a FCC metal, we propose a simplified hemispherical octahedral shape for platinum NPs supported on CeO_2. Supplementary Figure 12 (A) confirms a typical truncated octahedral shape in the STEM image. Therefore, in this study, the platinum nanoparticles are modeled by a truncated octahedral shape, bound by <111> and <100> facets with the two lowest surface energies according to the DFT calculation [15, 16]. The supported nanoparticles are then modeled by the upper half of the truncated octahedral. The fractions are displayed as a function of the NP diameter in Supplementary Figure 12 (B),

As discussed in Section S2.1, nanoscale STEM images inherently carry localized information, whereas photoemission spectra provide area-averaged data in the hundreds of micrometer scale. To minimize potential spatial resolution bias, we acquired STEM images from multiple regions, including low magnification overviews in Supplementary Figures 1-2, to better reflect the overall distribution and morphology of Pt NPs across the sample. On the other hand, platinum NPs can dynamically change their configurations under the reaction conditions. Recent advances in in situ electron microscopy with deep denoising reveal that upon CO exposure, Pt particles gradually deviate from their thermodynamically favored Winterbottom-like shape[17]. This morphological evolution is likely driven by local variations in Pt surface energy induced by Pt-CO strong interaction and changes in CO coverage, resulting in highly mobile atoms/clusters[11, 18]. During O_2–H_2 pretreatment, surface atom migration on the catalyst has been observed[19]. Under CO oxidation reaction conditions, in situ TEM images display pronounced motion artifacts and features attributable to particle mobility[11]. However, under saturated CO coverage, the Pt NPs undergo reconstruction

and still exhibits a truncated octahedron shape[20]. In inert N_2 atmosphere, the dynamic behavior of Pt is significantly suppressed compared to that in CO[11]. Notably, under typical water-gas shift conditions (i.e., co-feeding H_2O), Pt nanoparticles become considerably more stable, with only peripheral atoms near the cluster edge exhibiting mobility[18].

To demonstrate the Pt NPs shape and stability under relevant conditions, we performed in situ STEM imaging of the powder catalyst sample (NP_0) using a gas-cell holder (DENSsolutions Climate GVB) under two different sets of CO_2 + H_2 environments: 0.2 mbar CO_2 + 0.2 mbar H_2 at 250 °C and 0.25 bar CO_2 + 0.75 bar H_2 at 300 °C, as shown in Supplementary Figure 13 and Supplementary Movies 1–2. These conditions were chosen to bracket the pressure range from milli- to sub-bar levels, thus allowing the assessment of Pt NP behavior across relevant catalytic regimes. Due to the highly porous and high–surface area nature of the catalyst, projection images acquired under gas-cell conditions exhibit complex contrast: multiple voids, pores, and particle layers contribute to signal overlap and Z-contrast variation. As a result, direct atomic-resolution imaging of Pt was not achievable in these experiments. Nevertheless, dynamic sequences were successfully recorded at a frame rate of ~0.8 seconds, allowing us to track the temporal evolution of Pt nanoparticles with sufficient temporal resolution for reaction-relevant interpretations. Across both pressure regimes, the Pt nanoparticles maintained their size and morphology over several minutes of continuous imaging, showing no evidence of redispersion, sintering, or collapse. Although it is possible that finer structural dynamics (e.g., surface atom migration) may occur above our temporal and spatial resolution, the observed stability strongly supports the notion that on the 1–10 minute timescale relevant for operando XPS (e.g., Pt 4f acquisition typically spans ~10 min), the NPs exist in a structurally stable state. This set of experiments also confirms that Pt NP behavior is qualitatively consistent across mbar- to sub-bar pressure conditions. Despite limitations in spatial resolution, these real-time observations provide crucial experimental evidence that the structural assignment used in XPS interpretation—based on stable NP configurations—is physically meaningful under realistic gas environments.

Supplementary Figure 14 and Supplementary Movie 3 present a time-resolved series of atomic-resolution HAADF-STEM images (A–D) of a Pt nanoparticle supported on

CeO_2, along with the corresponding Fast Fourier Transforms (E–H). These snapshots, acquired at 00:00, 00:35, 01:42, and 02:50, respectively, allow direct visualization of the NP's orientation dynamics during observation. At t=00:00 (A), the particle displays a projected morphology with nearly hexagonal symmetry and well-defined lattice fringes, as indicated by the overlaid structural model. The corresponding FFT (E) reveals features that approximate a threefold symmetry, which resembles that of a [111] zone axis in face-centered cubic (fcc) Pt. While neither the real-space image nor the FFT exhibits a perfect [111] projection, this orientation serves as a reasonable approximation. At t=00:35 (B) and more clearly at t=01:42 (C), the particle undergoes a significant rotational change, and the originally visible atomic columns become indistinguishable. The corresponding FFT pattern (G) also evolves from a nearly threefold symmetric pattern into a rectangular distribution, indicating a deviation from the [111] zone axis. The FFT shows features that are more characteristic of a projection close to the [101] zone axis. The overlaid structural model and its FFT further support this interpretation. Although the nanoparticle is not perfectly aligned with the [101] zone axis, the observed image and FFT features are consistent with a reorientation from [111] to [101] (tilting angle between [111] and [101] = 35.26°). At t = 02:50 (D), the diffraction pattern (H) stabilizes, and the real-space fringes no longer evolve, indicating that the particle has reached a new steady-state configuration. Throughout the sequence, the particle retains a truncated-octahedral-like shape without signs of sintering, facet disorder, or significant atomic rearrangement. This sequence demonstrates a structurally coherent and directional rotation of the Pt NP from [111] to [101], without morphological collapse. The transformation is likely driven by interfacial energy minimization or interaction with the CeO_2 surface. Importantly, this reorientation appears to occur via a rigid-body rotation rather than surface diffusion or reconstruction.

Moreover, both empirical calculations and theoretical predictions provide statistical evidence for the prevalence of a hemispherical octahedral shape[13]. Notably, the nanoparticles with the smaller dimensions (<2.5 nm) exhibit a truncated octahedral morphology, characterized by 111 and 100 facets. This observation aligns with the thermodynamic principle of lowest-energy configuration, independent of the synthesis method employed[21]. Remarkably, this size range corresponds to most NPs investigated in this study. Atomistic simulations involving both molecular dynamics and

an empirical square-root bond-cutting model with Boltzmann statistics offer a comprehensive explanation.

The diverse interaction dynamics between platinum NPs and the various CeO_2 support facets can also result in different shapes of NPs[22]. In Supplementary Figure 19, we simulate the interaction of platinum with CeO_2 support to varying degrees based on the model of truncated octahedron, demonstrating how stronger interactions flatten the nanoparticles while weaker interactions lead to more diamond-like shapes. Therefore, for different facets of CeO_2, the interaction strength with Pt is likely to be different, thus changing the shape of the NP, but not affecting the trend of the key sites on the NP such as the fraction of corner atoms.

Our approach utilizes theoretically simulated NPs structures and empirical evidence that align with the observed behaviors of similar systems under comparable conditions. While the exact shape of each nanoparticle cannot be definitively ascertained, the use of a statistically prevalent model like the truncated octahedron provides a meaningful and reliable representation for understanding and predicting the catalytic behavior of these systems.

S1.3 DFT calculation of the effect of sodium on the structure evolution of platinum

Supplementary Figure 10 shows the differences in structures and adsorption energy of PtO_x species on the CeO_2 (223) surface with and without NaOH species. On the CeO_2 surface without NaOH species, the platinum atoms adsorb on the bridge site between two surface oxygen atoms. Platinum in the PtO and PtO_2 species are coordinated by four oxygen atoms, forming stable square planar [PtO_4] units[23]. In the case of PtO, one oxygen atom in the [PtO4] unit originates from a neighboring surface O site, leaving an oxygen vacancy site on the surface. In the presence of NaOH species, the structures of PtO_x species on CeO_2 surfaces do not change. PtO and PtO_2 interact with NaOH by forming a Na-O bond. In the oxidizing environment, platinum exists in the form of PtO and PtO_2 species. DFT calculations show that these species are more stable (0.45 eV for PtO and 0.57 eV for PtO_2) on the CeO_2 surface in the presence of NaOH species. NaOH will thus promote the dispersion of nanoparticles to form AD platinum under oxidative conditions, in agreement with the experimental findings. In the WGSR environment, the PtO and PtO_2 species will be reduced to atomic Pt species. The experimental results show that sodium species promote the sintering of AD platinum, forming larger platinum nanoparticles. However, the DFT calculations show that the NaOH species has a negligible influence on the adsorption of reduced platinum species. This implies that the sintering process cannot be explained simply by thermodynamic stability, but instead that other kinetic factors may play an important role in the sintering process.

S1.4 Different theoretical models of Pt NPs

Platinum NPs may have different shapes, depending on their size, position, interaction with the support, etc. This will lead to different fractions assigned to the corner atoms. On the other hand, the participation of ceria in WGS will place more emphasis on the role of the sites at the interface. We, therefore, also took into account the fraction of perimeter atoms, and two new theoretical physical models of the NP were established: "pancake" and "diamond". Supplementary Figure 19 (A) and (B) show the two models and the fraction of species as a function of size. The pancake model is flatter compared to the truncated octahedron due to the stronger Pt-CeO_2 interaction; in contrast, the diamond model represents the case of the weaker interaction, with a shape close to a diamond or a truncated bipyramid[24, 25]. Supplementary Figure 19 (C) presents a comparison of the size dependence of the calculated corner ratios combining both models together with the truncated octahedron model. The corner site shows similar fractions in the three models. Supplementary Figure 19 (D) shows that the fractions of the perimeter site are different in the three models, but none of them has a drastic drop as the size of the nanoparticle decreases. Thus, difference in NP shape has a minor effect on the rate of decay of the corner atoms; the fraction of perimeter atoms also cannot provide a faster drop.

S1.5 APXPS investigations of *Ce 3d* during the WGS reaction

The *Ce 3d* spectra in Supplementary Figure 21 were collected under the same reaction conditions as for *Pt 4f* spectra; and Supplementary Figure 22 displays the structural evolution with temperature, in CO+H_2O on different samples. In addition to the six samples loaded with platinum, the bare ceria support, prepared according to the method described in the main manuscript (Methods), was also tested as a reference. CeO_2 is reduced to a different extent on all samples. The bar plots of all samples show a consistent pattern during cooling: The fraction of Ce^{3+} is higher at lower temperatures. According to the activity test results, the catalyst has no WGS activity at 100°C; at 300°C, when high conversion is measured, a decrease in Ce^{3+} indicates that oxidation state of ceria surface has relevant modifications upon WGS reaction. Such a difference at 100°C and 300°C suggests that the WGS reaction involves the participation of ceria. This result can be verified further by the behavior of the AD_0 sample. AD_0 fresh is almost inactive and shows the lowest percentage of Ce^{3+} at low temperatures (100 and 250°C). As soon as AD Pt^{2+} sites sinter at 300°C, the fraction of Ce^{3+} increases and becomes comparable to that observed in the other catalysts. Metallic platinum boosts the local reduction of ceria, creating Ce^{3+} active sites for the reaction. The affinity of the reactants for different surface sites, i.e., CO always adsorbs on Pt while water adsorbs on ceria, places more emphasis on the importance of the Pt-ceria interface for WGS[26, 27].

As suggested by the dashed line in Supplementary Figure 22, at 100°C, when there is no observable conversion, the samples show a decreasing amount of Ce^{3+} from NP_0 to NP_5 and from AD_0 to AD_5, with bare-CeO_2 having the lowest percentage. To explain this, the Ce^{3+} fraction was plotted as a function of the size of Pt NPs (Supplementary Figure 23): As the size of NPs increases, the fraction of Ce^{3+} decreases. This may be related to the different magnitude of Pt-CeO_2 electron transfer[28, 29]. Small Pt clusters transfer more charge to the support, resulting in a greater reduction in CeO_2. However, several factors affect the CeO_2 valence state: Pt-CeO_2 charge transfer, reaction conditions, Na-loading and even the grain size of CeO_2. Moreover, the high (20-26%) Ce^{3+} fraction also indicates that 1 wt% Pt cannot provide so much charge. Therefore, we assume qualitatively that the lower Ce^{3+} percentage observed with the larger Pt NP is attributed, in part, to the weaker EMSI, which is consistent with the literature[29] and with the shift of *Pt 4f* to lower BEs.

S1.6 Mathematical expression of the kinetic model

The model assumes that two types of active sites, namely AC_1 and AC_2, possessing different activation energies E_1 and E_2, are dispersed on the surface of a catalyst. The proportion of AC_1 is x and the proportion of AC_2 is (1-x).

Overall activation energy is not the mere sum of E_1 and E_2; the reaction rate must be taken into consideration.

Arrhenius equation:

$$k = A \cdot exp(-E_a/RT) \quad (2)$$

Where:

- k is the rate constant,
- A is the pre-exponential factor,
- E_a is the activation energy,
- T is the absolute temperature (in Kelvin),
- R is the universal gas constant.

Differential form of Arrhenius equation:

$$E_a = RT^2 \frac{dlnk}{dT} \quad (3)$$

$$\frac{dk}{dT} = A \cdot exp\left(-\frac{E_a}{RT}\right) \cdot \frac{E_a}{RT^2} = \frac{kE_a}{RT^2} \quad (4)$$

Rate of AC_1 for WGS: the amount of CO converted from AC_1 in unit time

$$r_1 = k_1 \cdot [CO]^{O1} \cdot [H_2O]^{O2} \cdot [H_2]^{O3} \cdot [CO_2]^{O4} \quad (5)$$

Rate of AC_2 for WGS: the amount of CO converted from AC_2 in unit time

$$r_2 = k_2 \cdot [CO]^{O1} \cdot [H_2O]^{O2} \cdot [H_2]^{O3} \cdot [CO_2]^{O4} \quad (6)$$

where

- r is the WGS reaction rate,
- O is the reaction orders of reactants and products,
- Assuming that the reaction orders for AC_1 and AC_2 are identical.

The overall rate r_{eff} of AC_1 and AC_2 for the WGS reaction is:

$$r_{eff} = x \cdot r_1 + (1-x) \cdot r_2 \quad (7)$$

$$r_{eff} = (x \cdot k_1 - x \cdot k_2 + k_2) \cdot [CO]^{O1} \cdot [H_2O]^{O2} \cdot [H_2]^{O3} \cdot [CO_2]^{O4} \quad (8)$$

Overall rate constant k_{eff}

$$k_{eff} = x \cdot k_1 - x \cdot k_2 + k_2 \quad (9)$$

$$k_{eff} = x \cdot (k_1 - k_2) + k_2 \quad (10)$$

$$\ln k_{eff} = \ln [x \cdot (k_1 - k_2) + k_2] \quad (11)$$

$$\frac{dlnk_{eff}}{dT} = \frac{1}{x(k_1 - k_2) + k_2} \cdot \frac{d}{dT}[x(k_1 - k_2) + k_2] \quad (12)$$

$$\frac{dlnk_{eff}}{dT} = \frac{1}{k_{eff}} \cdot [x\left(\frac{dk_1}{dT} - \frac{dk_2}{dT}\right) + \frac{dk_2}{dT}] \quad (13)$$

$$\frac{dlnk_{eff}}{dT} = \frac{1}{k_{eff}} \cdot [x\left(\frac{k_1 \cdot E_1}{RT^2} - \frac{k_2 \cdot E_2}{RT^2}\right) + \frac{k_2 \cdot E_2}{RT^2}] \quad (14)$$

The expression for the overall activation energy E_{eff} is

$$E_{eff} = RT^2 \frac{dlnk}{dT} \quad (15)$$

$$E_{eff} = \frac{1}{k_{eff}} \cdot [x(k_1 \cdot E_1 - k_2 \cdot E_2) + k_2 \cdot E_2] \quad (16)$$

$$E_{eff} = \frac{x(k_1 \cdot E_1 - k_2 \cdot E_2) + k_2 \cdot E_2}{x \cdot (k_1 - k_2) + k_2} \quad (17)$$

$$E_{eff} = \frac{x(r_1 \cdot E_1 - r_2 \cdot E_2) + r_2 \cdot E_2}{x \cdot (r_1 - r_2) + r_2} \quad (18)$$

Equation (18) does not adequately describe the experimental activation energy data on the basis of the measured WGS rates (not shown here). The reason is that r_1 and r_2 are the function of temperature but our Arrhenius data were collected at different temperature ranges.

We therefore used a unified kinetic model that makes use of all the available kinetic data with a full range of temperatures and based on equation (8):

$$r_{eff} = [x \cdot k_1 + (1-x) \cdot k_2] \cdot [CO]^{O1} \cdot [H_2O]^{O2} \cdot [H_2]^{O3} \cdot [CO_2]^{O4} \quad (8)$$

$$r_{eff} = x \cdot A_1 \exp(-E_1/RT) + (1-x) \cdot A_2 \exp(-E_2/RT) \quad (19)$$

- Assuming that $[CO]^{O1} \cdot [H_2O]^{O2} \cdot [H_2]^{O3} \cdot [CO_2]^{O4}$ is a constant and merge it into the pre-exponential factor A_1 and A_2.
- Assuming that the structure of the platinum no longer changes in the Arrhenius test.

The experimental data r_{eff}, x and T (Supplementary Table 10) are substituted in the above equation to estimate A_1, A_2, E_1 and E_2. Experimental data were fitted in OriginPro using the Orthogonal Distance Regression(ODR) Algorithm. To cross-check, we also carried out a least-squares minimization in python using the scipy.optimize.curve_fit function. Both fitting results (Supplementary Table 11) are close, indicating that the fittings are reliable. By means of the fitted results, we calculated r_1 and r_2 at 250 and 300°C, respectively.

The activity difference of r_1 and r_2 is about 1380-fold at 250°C; hence CPS contributes almost all the conversion at low temperature. At 300°C, the gap narrows to about 410 fold, when the contribution of other sites to the activity starts to become significant, which is also the reason why E_a differs on different samples. The activity values calculated according to this model were compared with the measured results as a function of temperature (see Supplementary Figure 25). Because the fitting results by python and by OriginPro are close, only the results fitted by python are shown. We then took the average temperature in the Arrhenius plot for each sample in Figure 2(A) and 2(B) and estimated the activation energy at those specific temperatures by equation (18).

The calculated results are listed in Supplementary Table 12, and the predicted values of E_a are compared with the measured values in Figure 3(D).

In addition, we have included the original fitting data without the terrace peak shift to 72.2 eV on small NPs in Supplementary Figure 26. We have provided a comparison of the terrace and corner fractions before and after correction in Supplementary Table 13. Both the fitting details and the quantitative fractions demonstrate minimal modifications. Supplementary Figure 27 displays the corrections between WGS activity and CPS fraction, revealing that the structure-performance correlation was even better before correction. Furthermore, the uncorrected structural data can still effectively fit the kinetic mathematic model with a R^2 value of 0.95, as shown in Supplementary Figure 28. The intrinsic activity difference between CPS and other Pt sites at 250°C is re-calculated to be 973 times. These comparisons strongly support

the notion that this adjustment has minimal influence on our overall results, ensuring the robustness and reliability of our conclusions.

S1.7 Reaction energy analysis

Reaction energy calculations have been performed on two Pt NPs at sizes of 0.9 nm (Pt0.9) and 1.6 nm (Pt1.6), supported on the CeO_2 (111) surface. The number of Pt atoms are 25 and 86 respectively. Supplementary Figure 30 illustrates the atomic structures for the small (0.9 nm, naming Pt0.9) and large (1.6 nm, naming Pt1.6) Pt NP models on CeO_2 (111) slab, modeled by a p(6 × 6) unit cell and three atomic layers. Both NPs exhibit a truncated octahedral shape with four highlighted corners. The NPs were sampled across various (eight) positions and orientations on the surface, and the most stable geometries were selected for the reaction. Given the strong adsorption of CO on platinum particles, we assumed full CO coverage on Pt surface atoms that do not directly interact with the CeO_2 substrate. The surface chemistry of CeO_2 is inherently complex due to the presence of oxygen vacancies and possible hydroxyl (OH) group coverage. However, since our focus is on the *COOH dissociation process (as discussed in the following paragraph) we employed a clean CeO_2 surface to prevent the introduction of additional complexities unrelated to this reaction step.

Various reaction pathways, including the "redox" pathway[30, 31, 32] and the "associative" pathway[27, 33, 34], have been proposed for WGS mechanism. Our previous experimental findings revealed an associative reaction mechanism via carboxyl pathway on the same Pt/CeO_2 samples examined in the present study[35]. The rate-determining step is the dissociation of carboxyls (*COOH), which is in line with other theoretical calculations[27, 36]. Therefore, the carboxyl pathway was selected for our modeling, and we exclusively focused on this step in our analysis for the evaluation of activation energies based on the scaling relation between reaction energy and activation energy. The carboxyl pathway can be described by the following intermediate steps (IMs):

IM1: $*_{(Pt)} - *_{(O)} - *_{(Ce)} + CO_{(gas)} + H_2O_{(gas)}$

IM2: $CO_{(Pt)} - *_{(O)} - *_{(Ce)} + H_2O_{(gas)}$

IM3: $CO_{(Pt)} - *_{(O)} - H_2O_{(Ce)}$

IM4: $CO_{(Pt)} - H_{(O)} - HO_{(Ce)}$

IM5: $COOH_{Pt} - H_{O} - *_{Ce}$

IM6: $CO_{2(Pt)} - H_{(O)} - *_{(Ce)}$

IM7: $H_{(Pt)} - H_{(O)} - *_{Ce}$

IM8: $2H_{(Pt)} - *_{(O)} - *_{Ce}$

IM9: $*_{(Pt)} - *_{(O)} - *_{Ce}$

*COOH dissociation process (IM5→IM6) is the rate-limiting step of the carboxyl pathway, thus we focused calculations on this step.

Supplementary Figure 30 illustrates the atomic structures and reaction energies during *COOH dissociation on 0.9 nm and 1.6 nm Pt nanoparticles. To ensure statistical validity and representativeness, we analyzed one Pt site from each of the four corner locations, resulting in a total of four Pt sites. Reaction energies vary depending on the local environment of the active Pt sites. In Panel B4, we observed an unusually high reaction energy of 1.1 eV, attributed to the direct desorption of CO_2 following the dissociation of *COOH. Unlike other cases where CO_2 remains adsorbed on Pt, this energy is not directly comparable to other values and has not been included in the subsequent analysis. The mean and median reaction energies are 0.20 eV and 0.195 eV, respectively, for Pt0.9, compared to 0.52 eV and 0.44 eV for Pt1.6. Although activation energies cannot be directly averaged due to their impact on the reaction rate is via an exponential function, the distribution of reaction energies shown in Supplementary Figure 32(A) reveals a statistically significant difference between the two groups, with a p-value of 0.029 (one-tailed Mann-Whitney U test). The approximately 0.25-0.3 eV reaction energy difference in the RDS reflects a substantial difference in activation energies, based on the scaling relations. This aligns with the pronounced disparity in intrinsic activities between small and large Pt nanoparticles. The lower activation barrier for the 0.9 nm Pt nanoparticle is likely related to the adsorption energy of the reaction products. Since the reaction order of CO_2 was determined to be zero in the kinetic tests, we focused on the adsorption behavior of *H species, as H_2 exhibits a negative reaction order in both our kinetic results in Supplementary Figure 39 and previous literature[37]. For *H adsorption energy calculations, after obtaining the optimized geometry for the *COOH dissociation pathway, we removed the adsorbed CO_2 and re-optimized the structure to isolate the influence of *H adsorption. As highlighted in Supplementary Figure 32(B), the *H adsorption energy on Pt0.9 remains statistically significantly higher than that on Pt1.6, which is fully consistent with the observed difference in their reaction energies. To ensure completeness and transparency, we have also provided an analysis including the 1.1 eV value in Supplementary Figure 33, where the statistical significance remains.

S1.8 Bader charge analysis

Bader charge calculation has been performed on three Pt NPs at sizes of 0.9 nm (Pt0.9), 1.2 nm (Pt1.2), and 1.6 nm (Pt1.6), supported on the CeO_2 (111) surface. The number of Pt atoms are 25, 50 and 86 respectively.

In the literature, charge transfer from Pt to CeO_2 has been indicated by counting the number of Ce^{3+} cations[29]. In our calculation, there was no Ce^{3+} formation in all three Pt NP models, which indicates that Ce^{3+} cations are not stable in our models. This is because the CeO_2/Pt is a very complex system with many factors affecting the formation of Ce^{3+}, such as surface defects (oxygen vacancies and surface steps) and particle shapes. This is a well-known issue with DFT calculations, requiring special approaches to capture all these effects (see e.g. introduction of Ref. [38]).

Describing localized 4f states in Ce^{3+} accurately poses a well-known challenge in DFT due to self-interaction errors. Although the DFT+U method is widely used to address these issues, the result often depends strongly on the choice of the U parameter, which can vary among different studies and computational frameworks. A higher U value can indeed stabilize Ce^{3+} , but it may distort other material properties (e.g., reaction energies, lattice parameters) away from experimental benchmarks.

In our previous work[23], we found that U = 4.5–5.0 eV provides a balanced description of properties such as lattice constants, band gaps, and redox energetics for CeO_2. Our study of single Pt atoms and Ce^{3+} on various CeO_2 surfaces showed that U = 5 eV allowed electron donation from Pt only on the stepped (112) surface, while U = 8 eV stabilized Ce^{3+} more broadly but significantly altered material properties. For instance, the CeO_2 to Ce_2O_3 reduction energy dropped from 355 kJ/mol (U = 5 eV) to 259 kJ/mol (U = 8 eV), deviating from the experimental 388 kJ/mol. To avoid excessively large U values that compromise other physical properties, we adopt an alternative strategy: 4f-in-core pseudopotentials. By treating the 4f electron as part of the ionic core, one sidesteps many of the complications of partially filled f orbitals while still capturing the essential physics of Ce^{3+}sites. This method has been validated across multiple DFT codes (both plane-wave and Gaussian-type basis implementations like CP2K used in this work), ensuring robust descriptions of catalytic and redox phenomena without overly large U corrections.

In order to simulate Ce^{3+} cations and the charge transfer process, as previously performed by literature[39], we then proceeded by using a special norm-conserving 4f-in-core pseudopotential for the Ce^{3+} cations[38]. This practically enforces a maximum oxidation state of 3+ for the atoms with this 4f-in-core pseudopotential, since the 4f electron is in the core and cannot be removed. For each nanoparticle, we first optimized the geometries with normal Ce pseudopotentials. Then, we substituted one Ce atom on the top layer with the 4f-in-core pseudopotential and optimize the structure again. Finally, we could calculate the relative stability of this structure with respect to the structure with normal pseudopotential by:

$$\Delta E = (E_{slab\_4fpp} + E_{Ce2O3\_bulk}) - (E_{slab} + E_{Ce2O3\_bulk\_4fpp}) \quad (20)$$

Where E_{slab} is the energy of the surface slab with Pt nanoparticle, and E_{Ce2O3_bulk} is the energy of bulk Ce_2O_3, and 4fpp means one Ce atom is substituted by the 4f-in-core pseudopotential. By employing a 4f-in-core pseudopotential in the slab, we force the formation of a Ce^{3+} cation on the surface slab. Additionally, the energy of the bulk Ce_2O_3 is incorporated to cancel the absolute energy difference introduced by the two different pseudopotentials.

The ΔE is an indicator of the stability of creating a Ce^{3+} cation on the surface slab. We calculate the ΔE for slabs without Pt NP and slabs with different NPs. Supplementary Table 14 shows the stability of the Ce^{3+} cation on the CeO_2 surface with and without Pt NPs. The values are positive in all cases, indicating that the Ce^{3+} cations are not stable in our model, which is consistent with the result of our DFT calculation with normal pseudopotentials, as discussed above. However, the value decreased from 2.50 eV (for a pure CeO_2 surface) to a range of 0.37 to 0.47 eV (for a CeO_2 surface with a Pt NP on top). This indicates that Pt NPs play a significant role stabilizing the surface, thus facilitating the formation of Ce^{3+}. All our simulations considering different sizes of Pt NPs show similar relative stability when one Ce^{3+} is formed. We note that, even though in this model there is always one electron transferred from Pt to the CeO_2 support, the "charge transferred per Pt atom" in the 3D NP changes, due to the number of Pt atoms in the nanoparticle being different.

More importantly, we also performed a Bader charge analysis of perimeter atoms on the three NPs with and without the formation of one Ce^{3+} cation, i.e., averaging charge transfer among all perimeter atoms on the platinum NP to CeO_2. Our results, shown

in Supplementary Figure 34, indicate a clear difference in behavior between small NP (Pt0.9) and larger NPs(Pt1.2 and Pt1.6).

It is important to emphasize that the absolute numbers in Supplementary Figure 34 should not be directly interpreted, given the utilization of a pseudopotential approach within the CP2K code. This method does not yield the exact core charge distribution, unlike other pseudopotential DFT codes that employ PAW pseudopotentials capable of reconstructing the core charge. Consequently, minor constant shifts in Bader charge values may transpire. Nonetheless, the relative trends observed in the data remain indicative and predictive:

-With and without Ce^{3+}: the formation of a Ce^{3+} cation raises the mean Bader charge, shifting it towards less negative levels, suggesting enhanced charge transfer from Pt atoms to the support. These findings align with existing literature[29] as well as our own computations: upon totaling the Bader charges on all Pt atoms within the nanoparticle, a charge discrepancy of approximately $0.3e^-$ is observed, in line with alterations in the Bader charges on Ce from Ce^{4+} ($2.4e^-$) to Ce^{3+} ($2.07e^-$) as determined in bulk CeO_2 and Ce_2O_3.

-As a function of NP size: Both scenarios, whether with or without Ce^{3+}, demonstrate an enhanced charge transfer per platinum atom within small nanoparticles, thereby verifying our claims regarding the size dependence of Pt electronic structures.

S1.9 STEM-EELS analysis

In addition to theoretical calculations, a STEM-EELS analysis was conducted and detailed information on the sample preparation and the EELS experiments can be found in the Methods section (manuscript) and in Supplementary Figures 35-37, respectively. Supplementary Figure 36(A) and (B) illustrate that the EELS testing region is situated at the interface between Pt and CeO_2, in close proximity to the corner atoms of Pt NPs on CeO_2. When the Pt nanoparticles come into contact with the CeO_2 surface, the EMSI leads to the transfer of electrons at the metal/oxide interface[29]. These electrons are subsequently taken up by Ce^{4+}, resulting in their reduction to Ce^{3+}. Supplementary Figure 36(D) provides an example of the sensitivity of the Ce M edge to the oxidation state of CeO_2, as indicated by the increased area ratio of the M_5:M_4 peak, suggesting a reduced state[40, 41, 42]. A notable disparity in the oxidation state of CeO_2 supported by Pt nanoparticles of sizes 1.4 nm and 1.7 nm is observed, with the former exhibiting a higher M_5:M_4 ratio and thus a more reduced state. To further quantify the oxidation states of Ce in the vicinity of Pt nanoparticles of varying sizes, we employed the ratio of the peak areas of M_5 to M_4 as an indicator of the Ce^{3+} concentration[40, 41, 42], which is listed in Supplementary Table 15. Since the inelastic scattering mean free path of the core-loss EELS extends to ~100 nm at 300 keV[43, 44], the collected information is an average representation of the bulk. As such, we applied a correction to the M_5/M_4 ratio by considering the sample thickness. Additionally, in order to evaluate the charge transfer occurring at the Pt-CeO_2 interface, we further calibrated the M_5/M_4 ratio by taking into account the contact area of the interface, which is proportional to the square of the Pt NPs size. This correction accounts for an average charge transfer from a Pt atom to CeO_2.

To ensure that the morphology and dimensions of the Pt NPs were not affected by the electron beam during the EELS test, representative STEM images were captured both before and after the experiment, as shown in Supplementary Figure 37, revealing minimal changes in the platinum NPs dimensions. As previously discussed, the NPs can be highly dynamic with different snapshots during the EELS acquisition, and the long acquisition time (1s) suggests an averaged information for the snapshots.

Statistical analysis was performed on the data, taking into consideration the small sample size and the non-normal distribution. A non-parametric test, specifically the

Mann-Whitney U test[45], was chosen for analysis as it is suitable for comparing the medians of two independent samples. The Mann-Whitney U test yielded a p-value of 4.7×10^{-5}, well below the 0.001 threshold, indicating a significant difference between the two sets of data. Additionally, Welch's t-test[46] was performed, and the obtained p-value was approximately 8.6×10^{-10}. Given the robustness of these p-values, represented by three stars (***) in Figure 3(C), we have strong evidence to reject the null hypothesis that there is no difference between the two groups.

S1.10 Sodium-induced support modification

O 1s spectra and kinetic reaction order tests were used to demonstrate the Na-CeO_2 interaction, as well as its contribution to the WGS activity. Supplementary Figure 38(A) compares the H_2O and H_2 orders for AD_x samples, with detailed kinetic order test results shown in Supplementary Figure 39. The positive (H_2O) and negative (H_2) reaction orders indicate the promotion and inhibition effects of H_2O and H_2 towards WGS. Upon sodium addition, the H_2O order slightly decreases (from 0.9 to 0.7-0.8), while the H_2 order significantly increases (from -0.8 to -0.3), indicating that sodium can mitigate the inhibitory effect of H_2 on WGS[47]. Moreover, the small amount of sodium addition (AD_1) and large amount of sodium addition (AD_5) have the same effect on the reaction order of H_2, in line with the results for Na-Pt/rutile in ref.[37], implying that:

1. The effect of sodium on the reaction order is independent on the Pt NP size. Despite a consistent increase in NP size from AD_0 to AD_5, the reaction order experiences only minimal alteration from AD_1 to AD_5. In ref.[37], platinum remains in metallic state throughout the WGS process in both Na-free and Na-load samples, and the changes in reaction orders are only attributed to the modifications made to the support properties.

2. A relatively low loading of Na (Pt: Na = 1:1 in AD_1) is sufficient to alter the reaction order, while excess sodium (AD_5) does not contribute to further changes. Sodium preferentially occupies the most relevant locations on active sites, i.e., CPS at the Pt-CeO_2 interface; excess sodium migrates to the support but has no effect on the reaction order.

O 1s raw spectra in Supplementary Figure 38(B) show the effect of sodium on ceria measured on AD_x samples. Such spectra were acquired together with *Pt 4f* and *Ce 3d*, with a photon energy of 825 eV, and were normalized to the main peak intensity, using *C 1s* (Supplementary Figure 40) as a reference to align the BE scale. The lattice peak[48] of CeO_2 (AD_0 at 529.6 eV) shifts towards lower binding energy by 0.4 eV upon sodium addition (AD_5 at 529.2 eV). On the one hand, the energy shift of *O 1s* can be related to the band bending in the presence of metallic platinum due to work function differences between platinum and Na-CeO_2[49, 50]. However, the observation that only the main peak shifts significantly after adding sodium, while the other components

remain relatively unchanged, suggests a more localized effect. Therefore, the small but convincing shift on the main peak supports the formation of the Ce-O-Na structure, and is due to the electronegativity difference between sodium and cerium: when the O-Na structure forms, the electron cloud density on O is larger than that of O-Ce, leading negative BE shift of the lattice oxygen peak. The metal-O-alkali structure has already been reported in the literature[51, 52, 53]. It is important to highlight that the lattice oxygen peak of *O 1s* undergoes a shift, rather than broadening. This observation strongly suggests a homogeneous structure of Ce-O-Na within the probing depth of XPS. The *O 1s* spectrum was acquired at a kinetic energy of approximately 300 eV, corresponding to an inelastic mean free path of approximately 7.7 Å on ceria. Accounting for the XPS acquisition geometry, the mean escape depth is estimated as 6.6 Å, which is less than two unit cells of ceria. Nearly 80% of the photoemission signal originates from this depth, with the remainder from 2x and 3x this value. This indicates a homogeneous sodium distribution within the first two layers of the support. The STEM analysis depicted in Supplementary Figure 7 shows no agglomeration of sodium, and the negligible signal of sodium in energy dispersive spectrometry (Supplementary Figure 41) further confirms its highly dispersed nature. Additionally, the absence of any peaks in the diffraction pattern in Supplementary Figure 42, beyond those of pure ceria, implies the lack of a distinct sodium-containing phase. These findings align well with those previously reported literature[52, 54, 55]. Although surface hydroxyls (OHs) are considered significant intermediates in the WGS reaction[27, 56, 57, 58] and alkali metals influence their fraction[57, 58, 59], the intensities of OH-related peaks (531-532 eV)[48] in Supplementary Figure 38(B) exhibit negligible differences. This lessens their importance as a descriptor of WGS activity. Hence, at lower Na-loadings (Pt:Na=1:1), Na initially migrates to the metal-support interface and forms Na-O-Ce structure, as evidenced by the slight shift of the O 1s main peak for AD_1. Such modification of ceria promotes WGS by mitigating the inhibition effect of H_2, in line with the literature[47]. By increasing Na-loading (Pt:Na=1:5), the ceria surface is completely covered with homogeneous Na-O-Ce layer, but this does not further alter the reaction order.

The above results contribute to a comprehensive understanding of the sodium effect: on one hand, sodium inhibits WGS by promoting the sintering of platinum; on the other hand, sodium enhances WGS through support modification. The overall effect of

sodium on WGS is inhibition, implying that the contributions from the modification of the platinum structure overwhelms those from the support modification. Assuming that the support-modification have a significant impact on the activity, the proportional relationship between activity and platinum structure is the result of compensating for support-modification. Considering that activity is proportional to the number of active sites, the support-modification and activity should also have a proportional relationship. However, it is evident that when the activity changes significantly (AD_1 vs AD_5), the changes in reaction order caused by the support-modification are not as apparent, indicating no proportional relationship. This justifies why the contribution of support-modification to the activity is negligible in the proportional correlation between activity and Pt structure. A parallel comparison of AD_x and NP_x supports a similar conclusion, as the addition of the same amount of sodium to AD_0 and NP_0 results in markedly different activities. The strong correlation between WGS activity and platinum structure suggests that the platinum structure is the predominant descriptor for WGS activity.

S1.11 Compatibility analysis of APXPS data with activity tests by flow reactor to allow an *operando* analysis

Below is a discussion of the following three compatibility issues.

1) Activities in the APXPS chamber versus in the flow reactor

Supplementary Figure 43 compares the results of a fast activity test (Methods section in the manuscript) performed in the flow reactor with the hydrogen signal by means of MS during the APXPS experiment**.** The XPS cell is not a flow reactor; the catalyst pellet is different for each catalyst in terms of the amount of sample and pellet thickness (not a catalytic bed). However, the reaction can be followed quantitatively by following the production of hydrogen. Therefore, it is possible to make only qualitative comparisons across the samples.

On the other hand, it is relevant to compare the hydrogen signals for the same sample at different temperatures. As the temperature increases to the working temperature window of the catalyst, the hydrogen signal increases, indicating that the catalyst is functioning; the hydrogen signal at 300 °C is higher than that at 250 °C, in line with the results measured by the flow reactor. The red and black curves indicate good agreement. For example, they show the activation behavior of the AD_0 sample at 300 °C and the low CO conversion, reflected by the low H_2 production of the AD_5 sample at 300 °C.

Based on the equivalence of partial pressure levels experienced by the samples in both the XPS chamber and the flow reactor, as well as the strong correlation observed in the conversion and production data, we put forth the suggestion that this combination provides a viable means for conducting *operando* analyses.

2) Pt structures during *operando* XPS in $CO+H_2O$ versus in $CO+H_2O+H_2+CO_2$ during Arrhenius measurement

APXPS experiments were carried out in $CO+H_2O$, while the Arrhenius data were acquired by co-dosing reagents and products ($CO+H_2O+CO_2+H_2$). The pretreatment of the samples is also different: Before data acquisition in $CO+H_2O$, the samples were heated in 0.1 mbar CO + 0.3 mbar H_2O up to 300 °C then cooled down to 250 °C before measurement. In the case of $CO+H_2O+CO_2+H_2$, the samples were heated in 0.1 mbar CO + 0.3 mbar H_2O up to 500 °C then cooled down to 250 °C and measured after switching to $CO+H_2O+CO_2+H_2$. To evaluate whether the spectroscopic data can

be directly compared to the results of the Arrhenius tests, we compared the *Pt 4f* spectra of NP_0 and NP_5 samples acquired at 250 °C in 0.1 mbar CO + 0.3 mbar H_2O and 0.1 mbar CO + 0.3 mbar H_2O + 0.1 mbar CO_2+ 0.5 mbar H_2. Supplementary Figures 44-45 give *Pt 4f* spectra and Supplementary Table 17 lists the results of fitting. The differences between the two experimental conditions are minor, indicating that XPS data can describe the structural information of samples in their working state; thus the correlation of Arrhenius plots with spectroscopy data is relevant.

3) Industrial relevance of this work

To remove relatively low CO concentrations from a reformate gas, WGS is a chemical method that can be used as an alternative to preferential oxidation (PROX) and methanation. Typically, a WGS reactor is used in series with a PROX reactor to achieve CO concentrations lower than 10 ppm for hydrogen fuel cell applications[60], which are susceptible to carbon monoxide poisoning. Unfortunately, the commercial WGS catalysts are unsuitable for fuel cell applications. The development of water gas shift catalysts for the application in fuel cell technology is of current interest due to the demand for clean fuel and the critical role of the water gas shift reaction in hydrogen fuel cells. Therefore, the conditions we applied in the flow reactor (100 ppm CO+ 300 ppm H_2O) and in the APXPS (0.1 mbar CO+ 0.3 mbar H_2O) are industrially relevant. A comprehensive understanding of the structure-activity relationship under such conditions will finally enable the rational design of WGS catalysts.

S1.12 Discussion of contrasting results in the literature with regard to identification of the active site

Our results provide a complete characterization of the structure-activity relationship, based on interface-sensitive spectroscopy and reactivity data, with the support of NPs model simulations. The following points summarize the main disagreements with regard to previously reported data, in an attempt to highlight the novelty and strength of this work.

1) As suggested by APXPS, the corner/CPS sites represent low fractions among the platinum species. This weak feature is hard to estimate by bulk-sensitive techniques such as X-ray Absorption Spectroscopy (XAS). Previous studies support this claim, showing that samples consisting exclusively of AD species and those with coexisting AD and small clusters showed qualitatively similar XAS features [61, 62].

2) Due to final state effects, the EMSI and electron transfer from Pt 5d to the CO molecule upon adsorption, the *Pt 4f* component assigned to corner sites is at a higher binding energy than that of a platinum foil reference. The binding energy is close to that of oxidized platinum and can be confused with AD (Pt^{2+}); for that reason some research groups describe it by the term $Pt^{\delta+}$ [29, 63].

3) AD Pt^{2+} may behave as a precursor for smaller clusters, formed upon sintering during the reaction (as demonstrated by our microscopy and XPS results), which possess more CPS sites and, thus, lower activation energy according to our prediction. This may explain why AD catalysts are usually considered more active than large NP catalysts.

S1.13 Discussion of the WGS performance of progressively deposited Pt on CeO_2(111)

Our results elucidate the exceptional WGS activity behavior observed in the model Pt/CeO_2(111) catalyst[28]. As platinum is progressively deposited onto the initially inactive CeO_2(111) surface, the WGS activity exhibits a volcano-shaped curve in correlation with Pt coverage, initially increasing and then decreasing. The peak activity occurs in catalysts containing Pt particles ranging from 1-2 nm in size. This phenomenon can be rationalized by our findings: as Pt is deposited, AD species initially form on the CeO_2(111) surface, contributing minimally to the WGS activity. However, when the fraction of small Pt clusters (<1.25 nm) emerges, the WGS conversion rate starts to increase, peaking with the maximum number of small clusters (<1.25 nm). Continued increases in Pt only promote the formation and increase of larger particles at the expense of smaller clusters (<1.25 nm), while the larger NPs contribute little activity due to the dramatic change in electronic structure effects. The absence of activity change for Pt coverages between 0.8 and 2.4 monolayers is likely attributed to the persistence of the main active sites, i.e., the few small clusters (<1.25 nm), while additional larger particles contribute marginally to the activity. This suggests that merely increasing the Pt loading (augmenting the number of clusters and sites) without controlling the size fails to enhance the number of active sites. Our conclusions also align with results obtained for gold (Au) deposited on Mo(112)-(8 × 2)-TiOx in the context of CO oxidation[64].

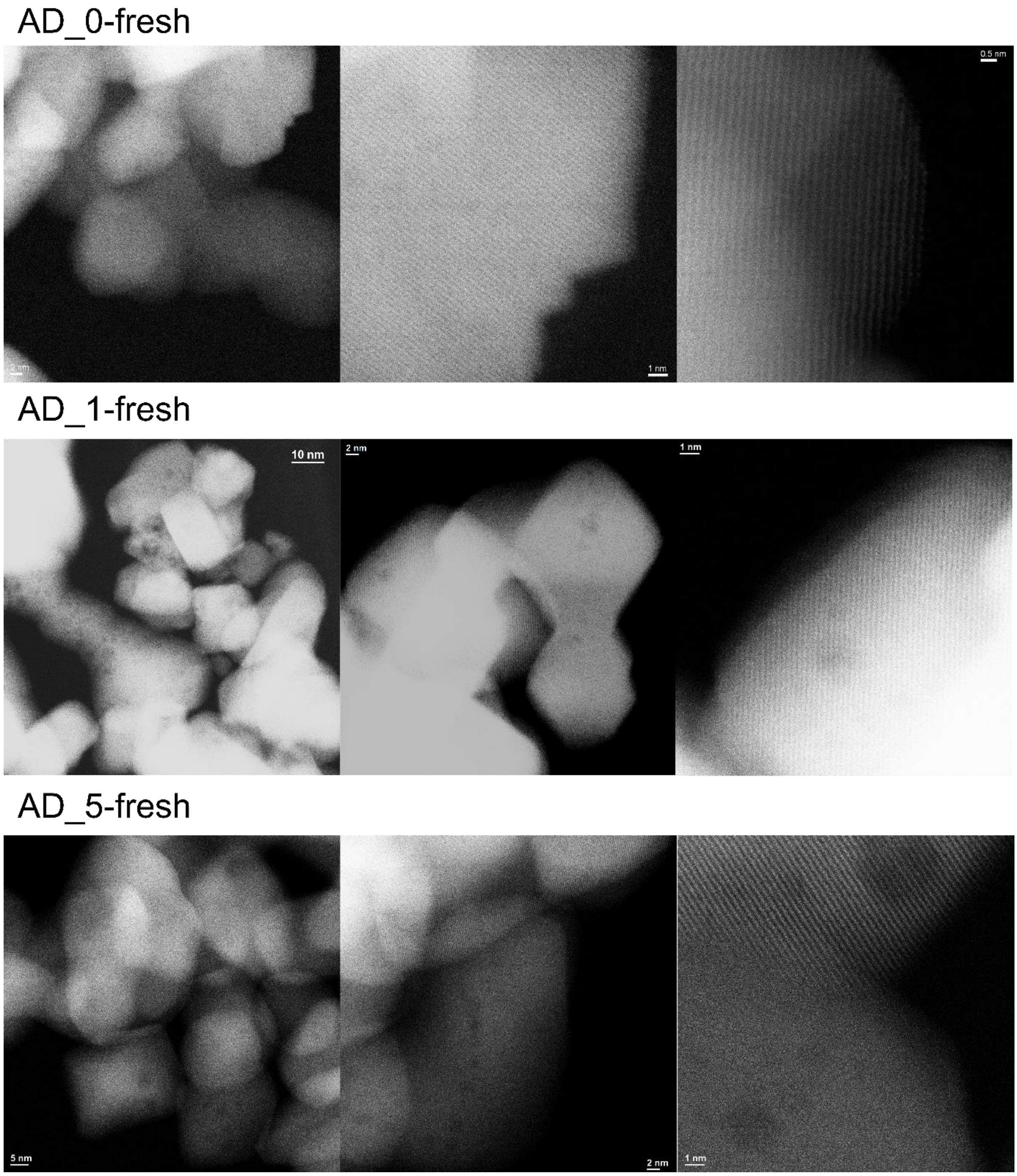

Supplementary Figure 1. Representative STEM images of the fresh AD_x catalysts.

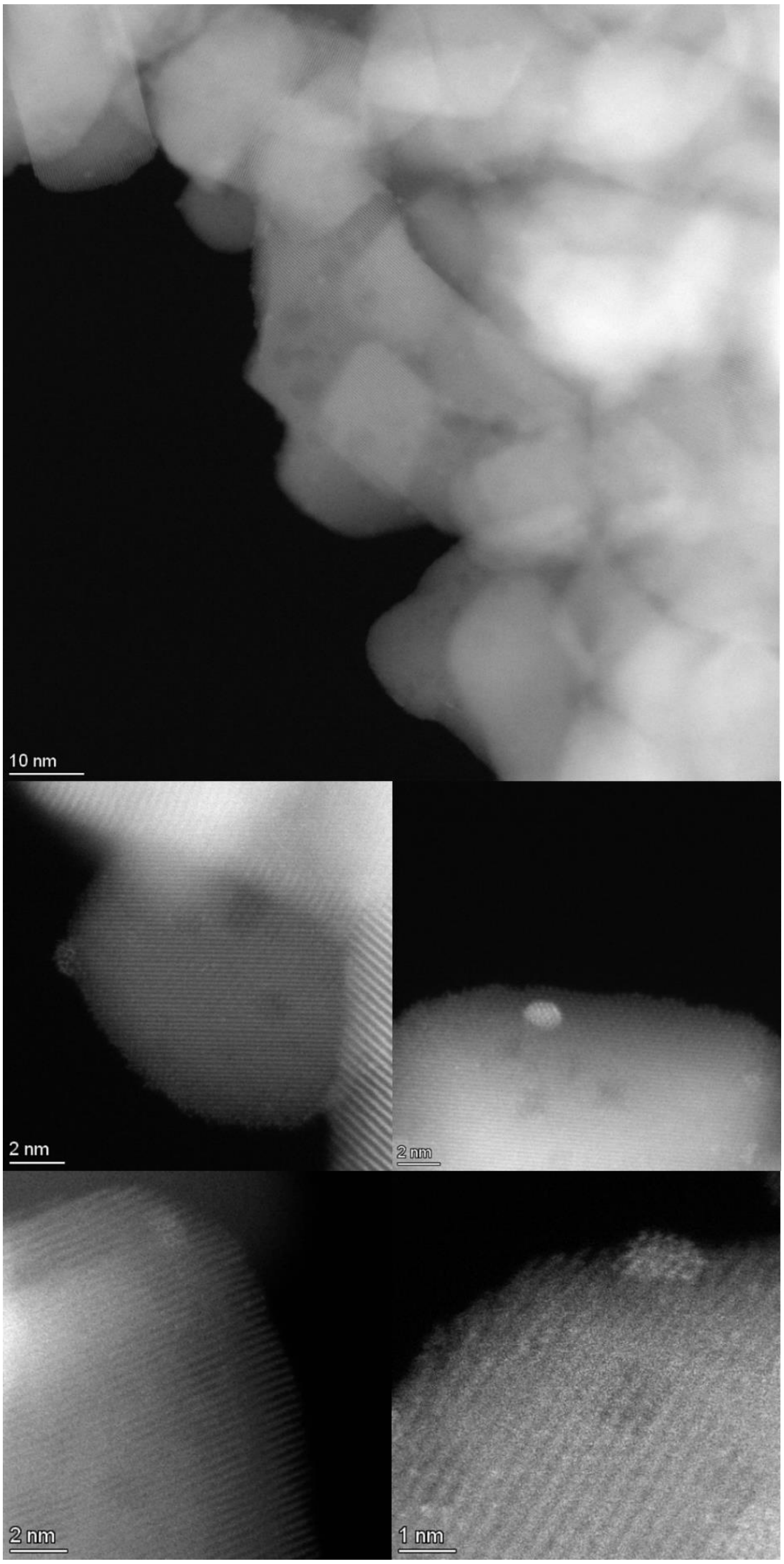

Supplementary Figure 2. High resolution HAADF-STEM images of used AD_1 at various magnification levels, presenting the co-existence of AD and NP species.

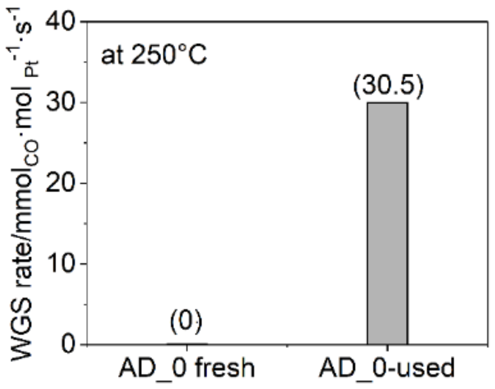

Supplementary Figure 3. WGS reaction rate of fresh/used AD_0 samples at 250°C calculated by the light-off conversion. Feeding: 100 ppm CO + 300 ppm H_2O, total flow=100 ml/min. Sample mass: 2mg catalyst and 200mg SiC.

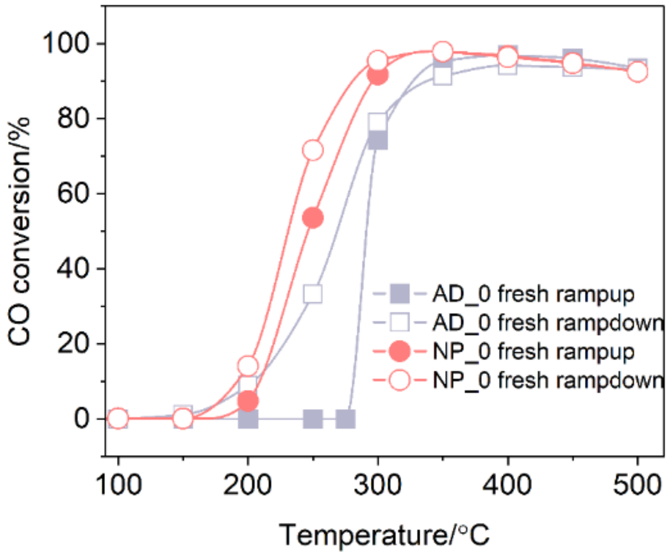

Supplementary Figure 4. Light-off curve of AD_0 and NP_0 at 100-500°C during ramp-up and ramp-down. Feeding: 100 ppm CO + 300 ppm H_2O, total flow=100 ml/min. Sample mass: 2mg catalyst and 200mg SiC.

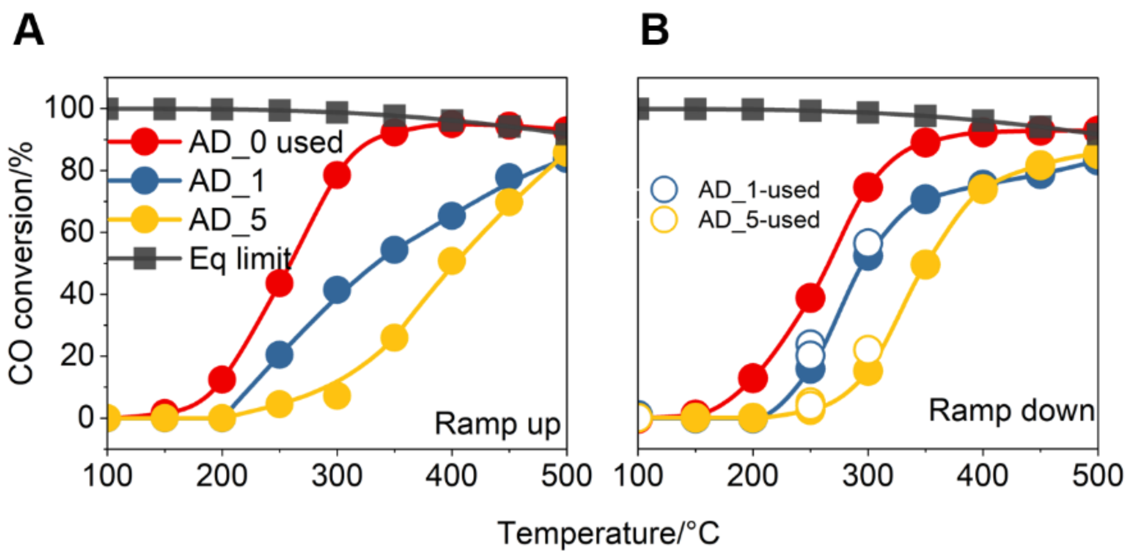

Supplementary Figure 5. Light-off curve of AD_x catalysts loaded with various amount of sodium at 100-500°C during (A) ramp-up and (B) ramp-down. To compare the activity of used AD_0 and AD_1/AD_5 samples, fast activity test results for the used AD_x samples after cool down are shown in empty circles in (B). Feeding: 100 ppm CO + 300 ppm H_2O, total flow=100 ml/min. Sample mass: 2mg catalyst and 200mg SiC.

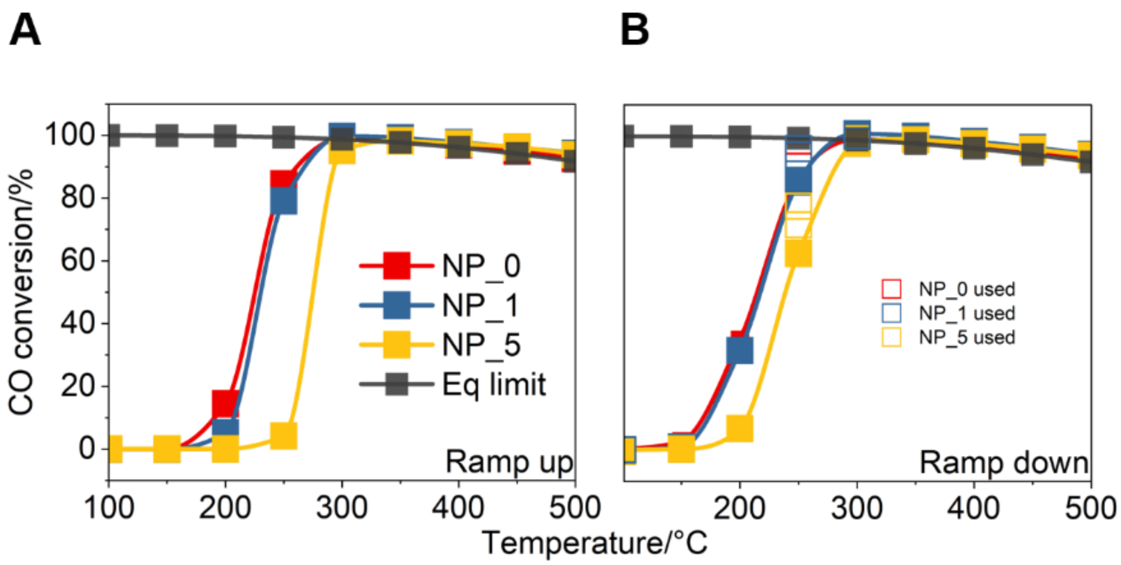

Supplementary Figure 6. Light-off curve of NP_x catalysts loaded with various amount of sodium at 100–500°C during (A) ramp-up and (B) ramp-down. To compare the activity of used NP_x samples, fast activity test results for the used NP_x samples after cool down are shown in empty squares in (B). Feeding: 100 ppm CO + 300 ppm H_2O, total flow=100 ml/min. Sample mass: 2mg catalyst and 200mg SiC.

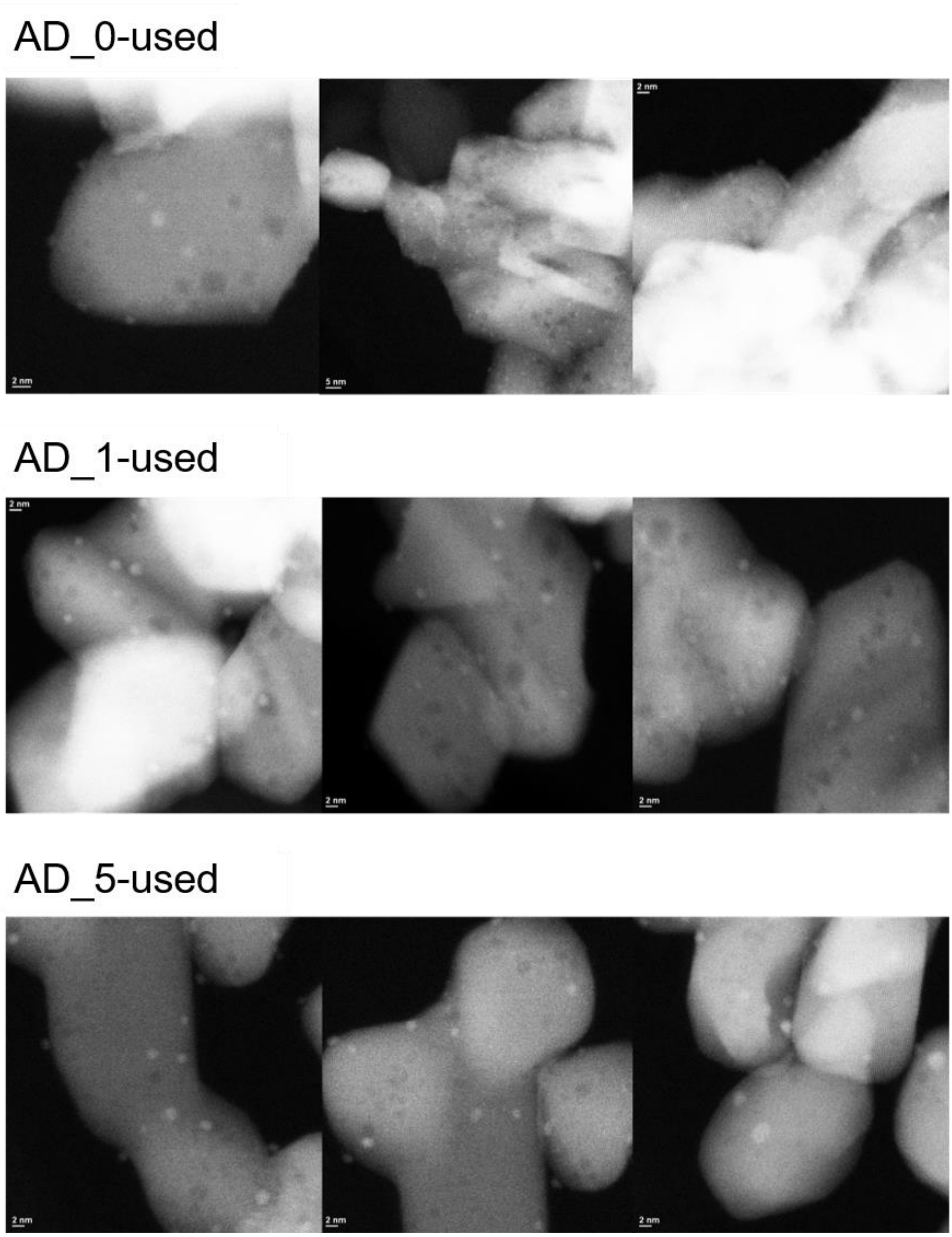

Supplementary Figure 7. Representative STEM images of the AD_x catalysts after WGS reaction.

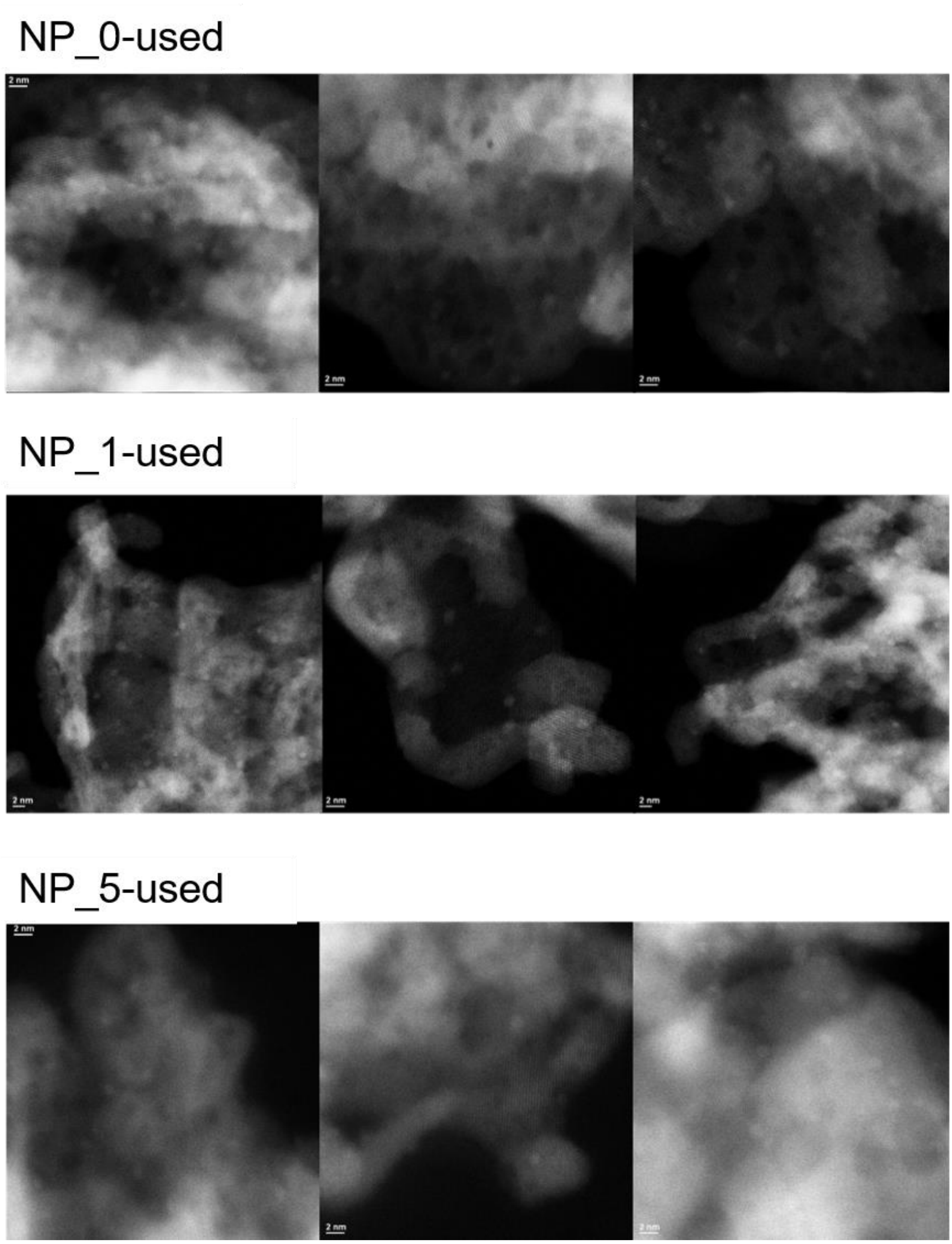

Supplementary Figure 8. Representative STEM images of the NP_x catalysts after WGS reaction.

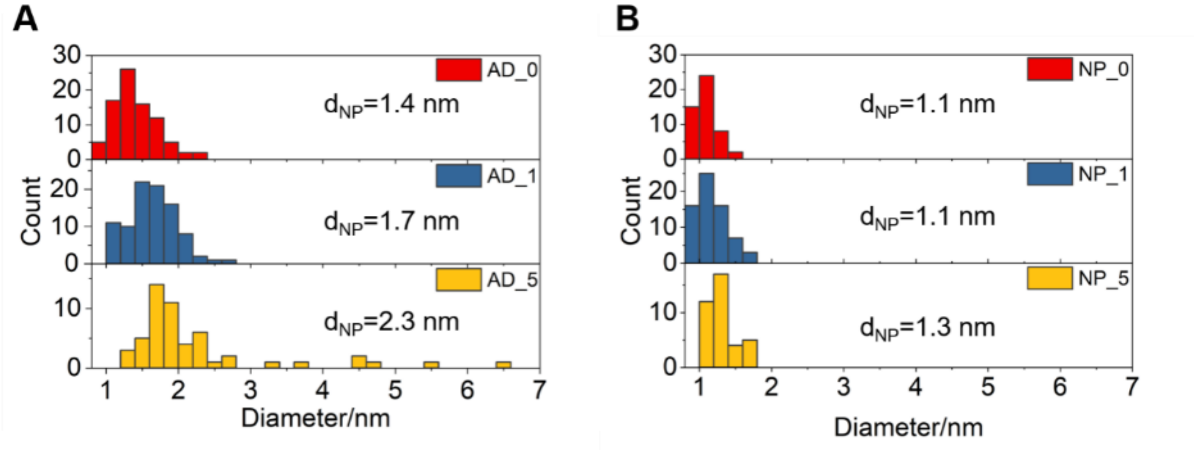

Supplementary Figure 9. Particle size distribution of the platinum nanoparticles on used AD_x samples (A) and used NP_x samples (B) obtained from the STEM images.

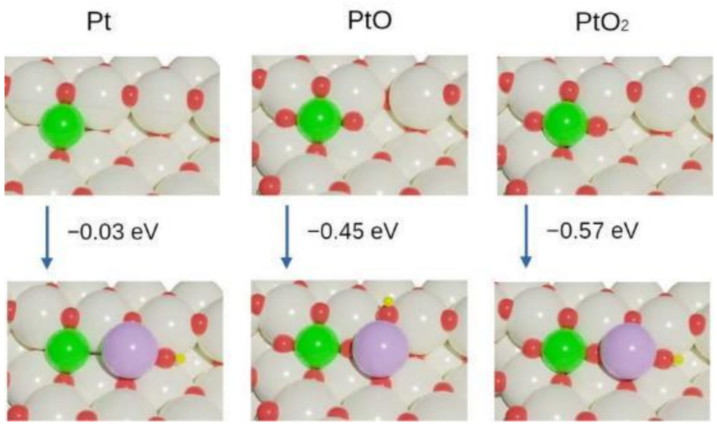

Supplementary Figure 10. Structures of PtO*x* species on CeO_2 (223) surface without and with NaOH species. Ce, O, Pt, Na and H atoms are shown as grey, red, green, purple and yellow spheres, respectively. The adsorption energy difference of PtO*x* species on CeO_2 (223) surface with and without NaOH species are also shown. Adsorption energies are calculated using Eq. (1).

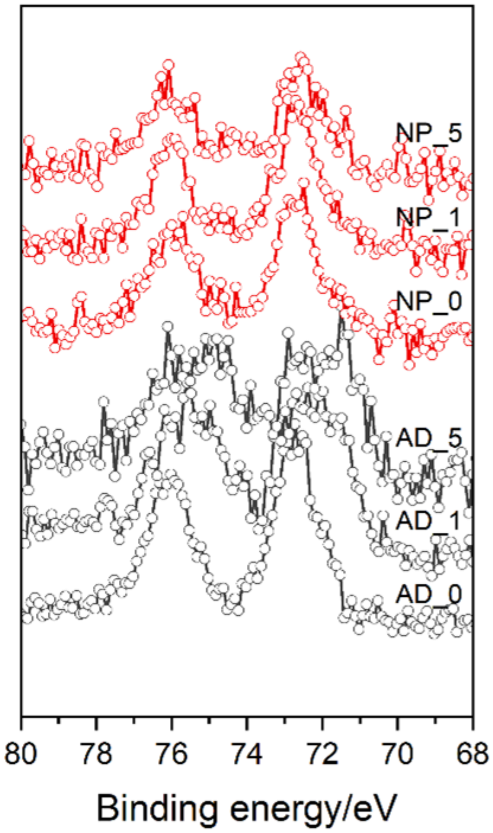

Supplementary Figure 11. Comparison of raw *Pt 4f* XP spectra collected at 250ºC in CO+H_2O (0.1+0.3 mbar) for AD_x and NP_x samples.

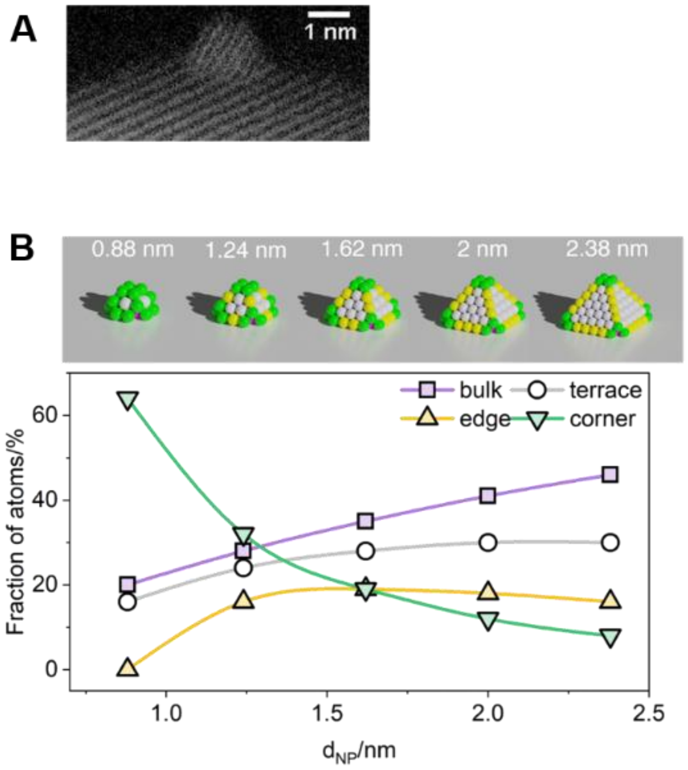

Supplementary Figure 12. (A) Typical STEM image of Pt NP for determining the particle shape in the model. (B)Illustrations of supported NP models (truncated octahedron) and atom fractions at different sites as a function of the NP model diameter.

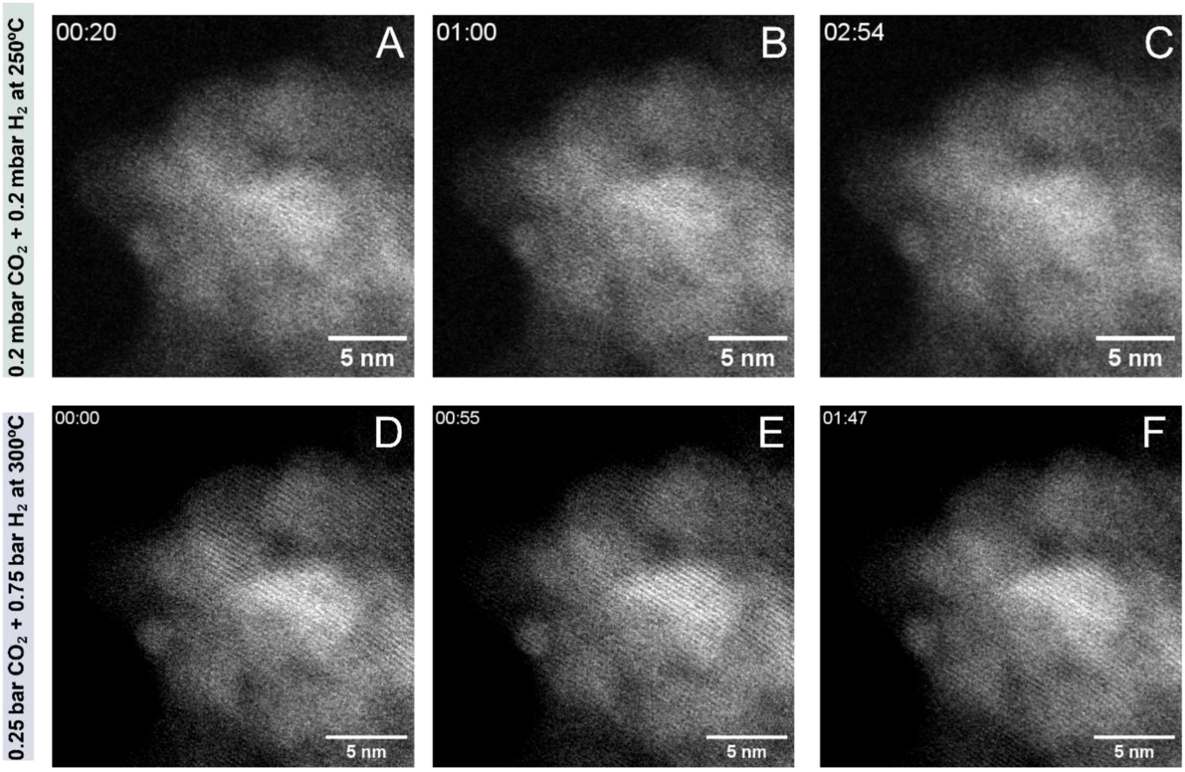

Supplementary Figure 13. Images of the NP_0 catalyst at 250 °C in 0.2 mbar CO_2 + 0.2 mbar H_2 (A-C) and at 300 °C in 0.25 bar CO_2 + 0.75 bar H_2 (D-F).

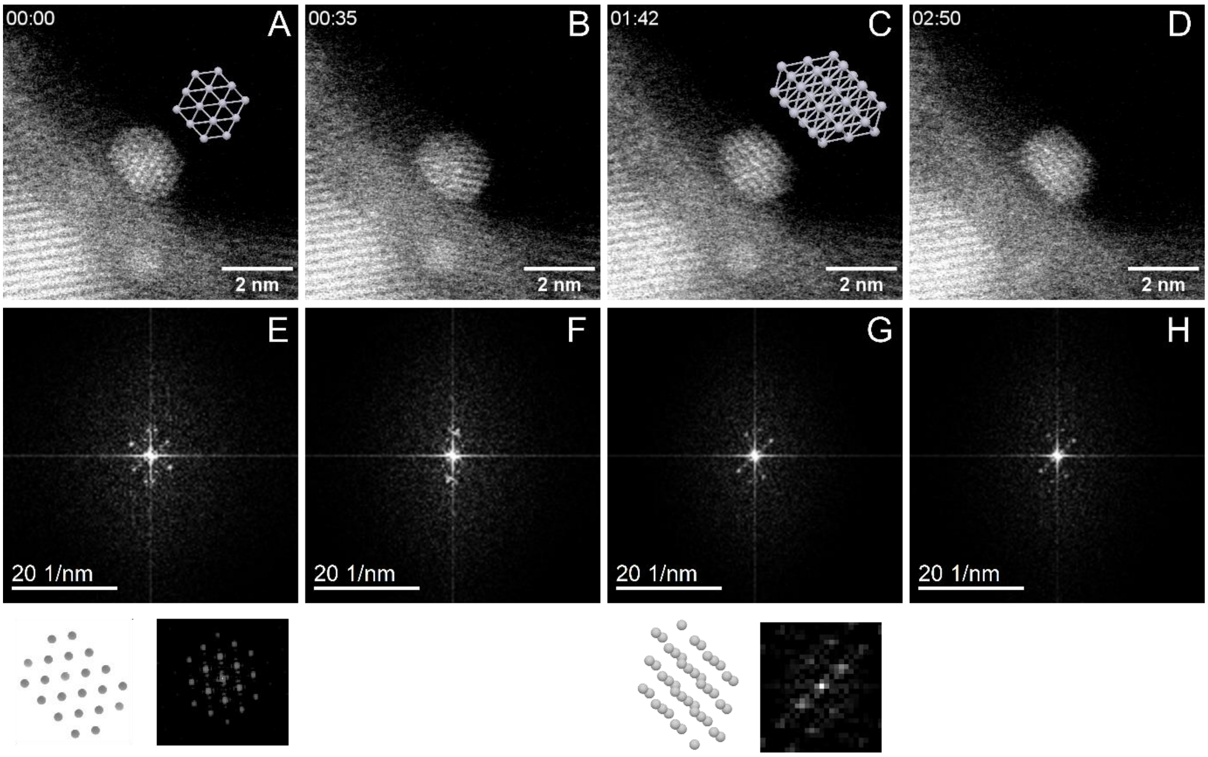

Supplementary Figure 14. Images of the model Pt/CeO_2 catalyst at 300 °C in 0.25 bar CO_2 + 0.75 bar H_2 (A-D) and the corresponding Fast Fourier Transform (FFT) of the Pt NP region. Panel E and G also show the FFT of ideal atomic models.

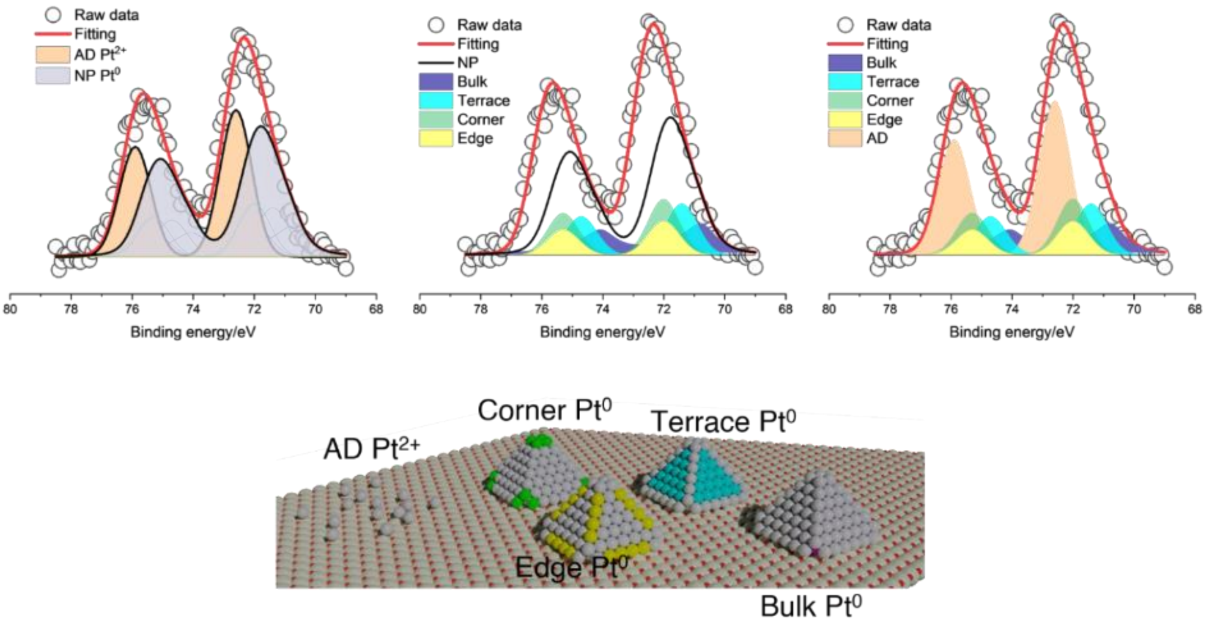

Supplementary Figure 15. An example of *Pt 4f* fitting details and the corresponding sites are highlighted with the same colors. Bulk Pt^0 atoms are in purple color but covered by the atoms on surface.

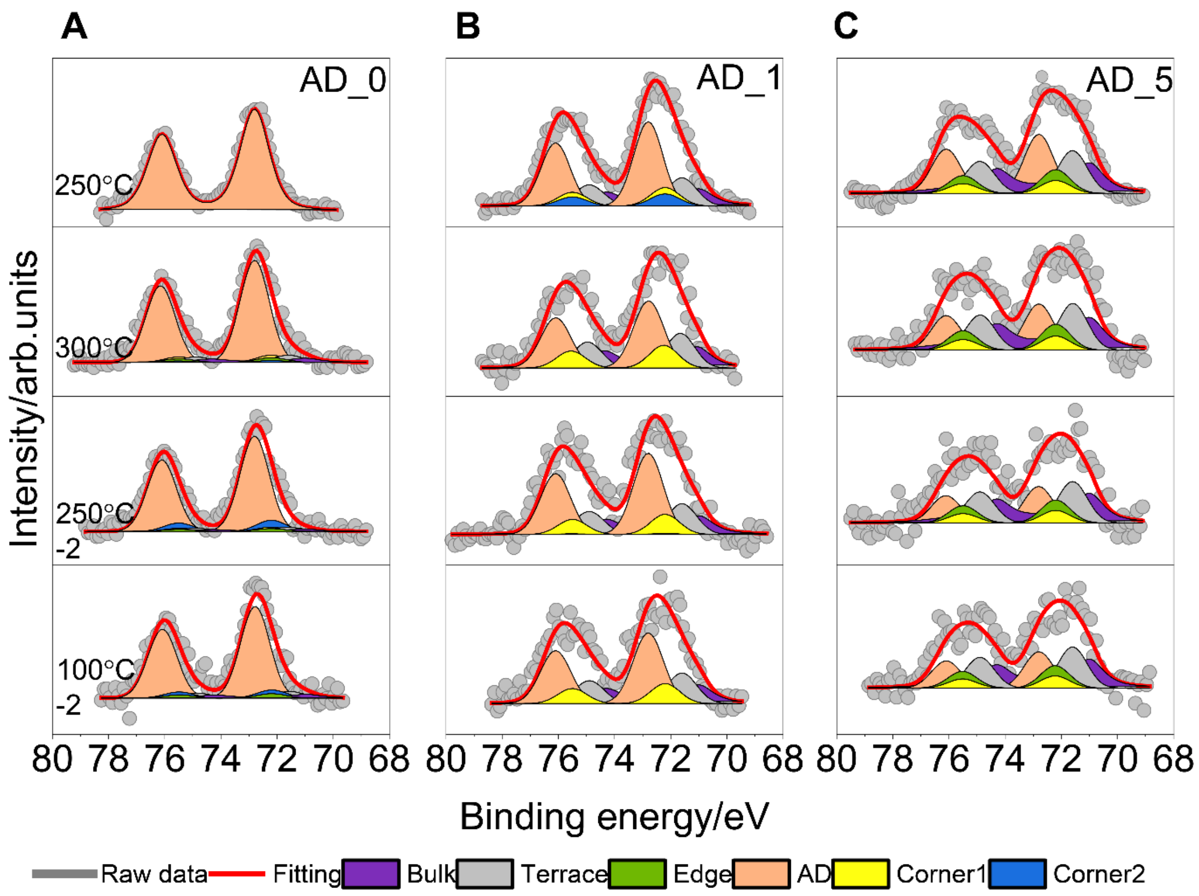

Supplementary Figure 16. Experimental X-ray photoelectron spectra and peak fitting of the *Pt 4f* region over AD_0 (A), AD_1 (B) and AD_5 (C) samples obtained in 0.1 mbar CO + 0.3 mbar H_2O, during ramp-up at 250-300°C and cool-down at 300-100°C, using 620 eV of photon energy.

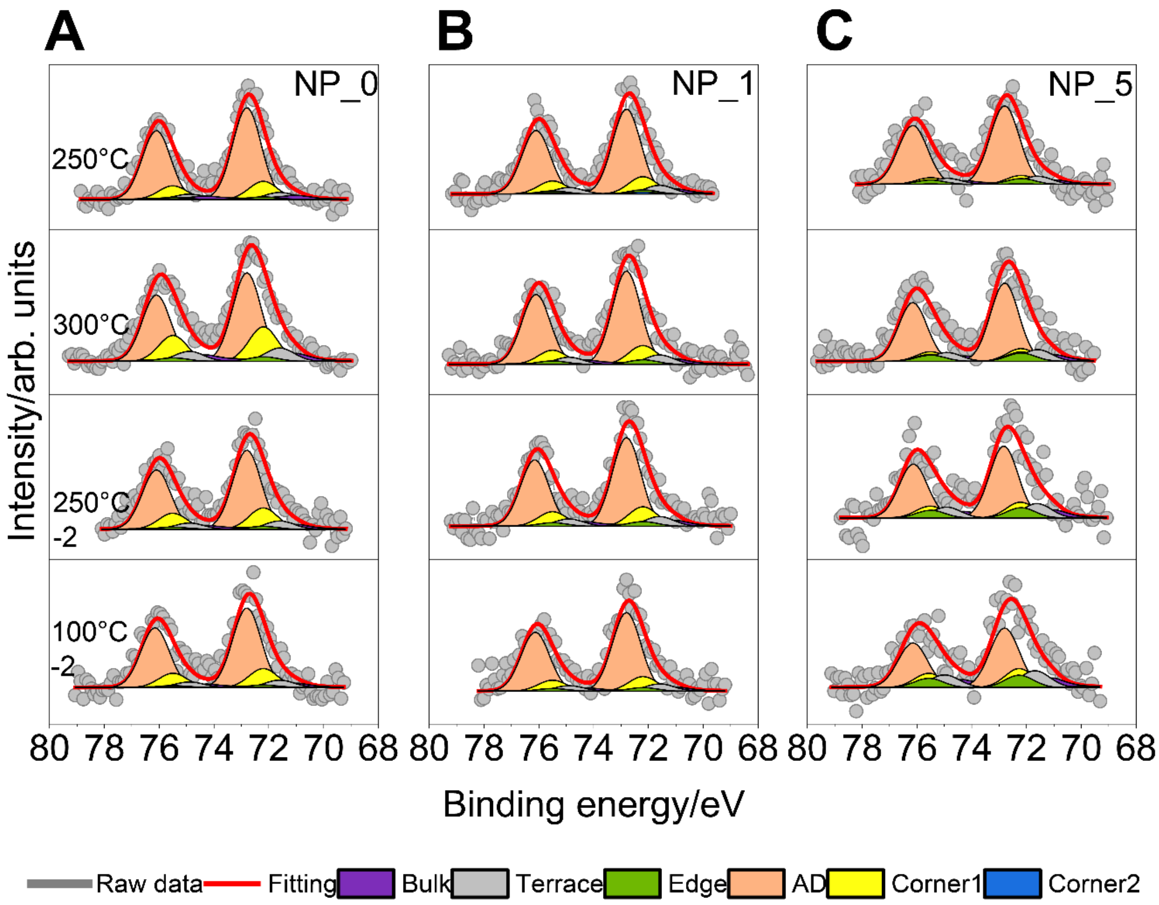

Supplementary Figure 17. Experimental X-ray photoelectron spectra and peak fitting of the *Pt 4f* region over NP_0 (A), NP_1 (B) and NP_5 (C) samples obtained in 0.1 mbar CO + 0.3 mbar H_2O, during ramp-up at 250-300°C and cool-down at 300-100°C, using 620 eV of photon energy.

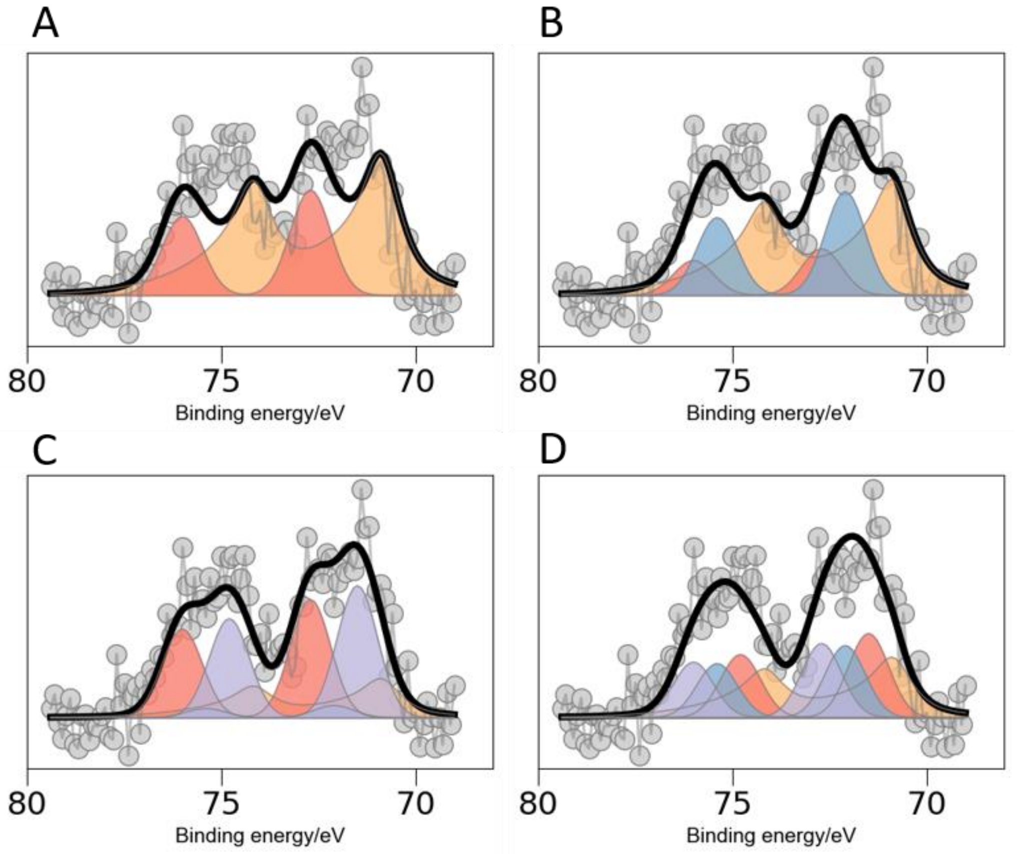

Supplementary Figure 18. Different strategies used to fit Pt 4f by (A) "traditional" double-peak arrangement (Pt^0 at ~71.0 eV and Pt^{2+} at ~72.8 eV), (B) three-peak arrangement that adds low coordination sites to the double-peak arrangement at 72.2 eV (C) four-peak arrangement that separates the corner/edge (72.2 eV) and terrace (71.6 eV) on the basis of three-peak arrangement but not constraining the peak area between them. (D) four-peak strategy used in this work but constraining the peak area between NP components according to Pt NP size.

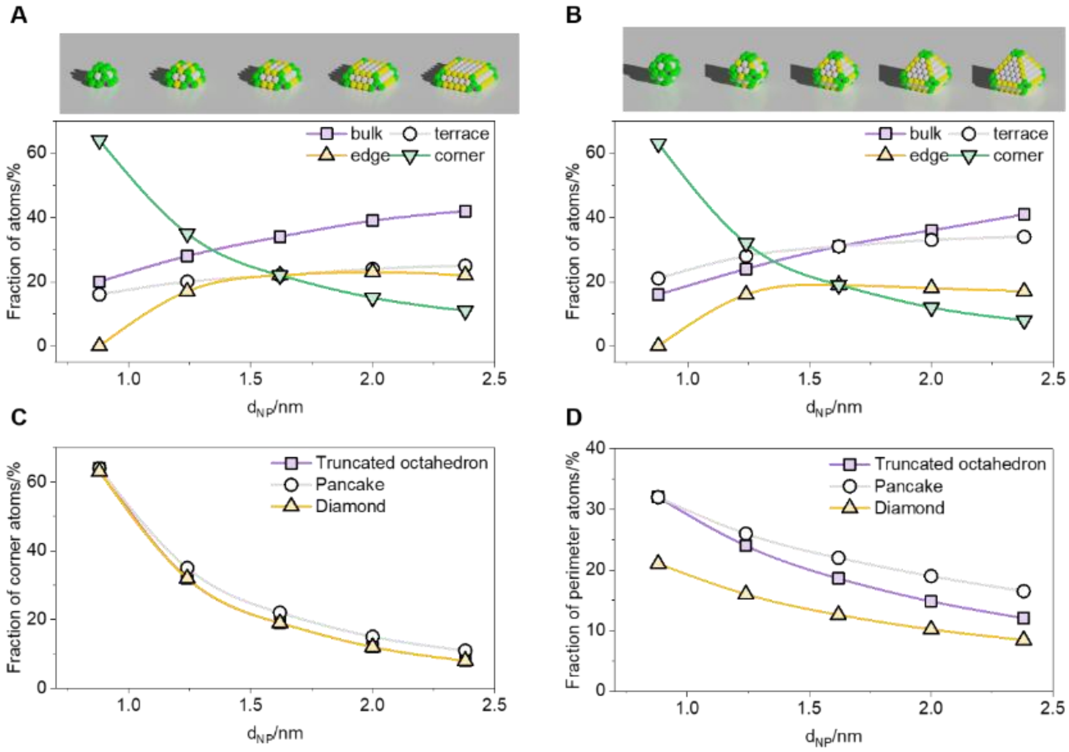

Supplementary Figure 19. Illustrations of supported NP models and atom fractions at different sites as a function of the NP model diameter. (A) "Pancake" and (B) "Diamond". Atom fractions of (C) corner and (D) perimeter sites as a function of the NP model diameter.

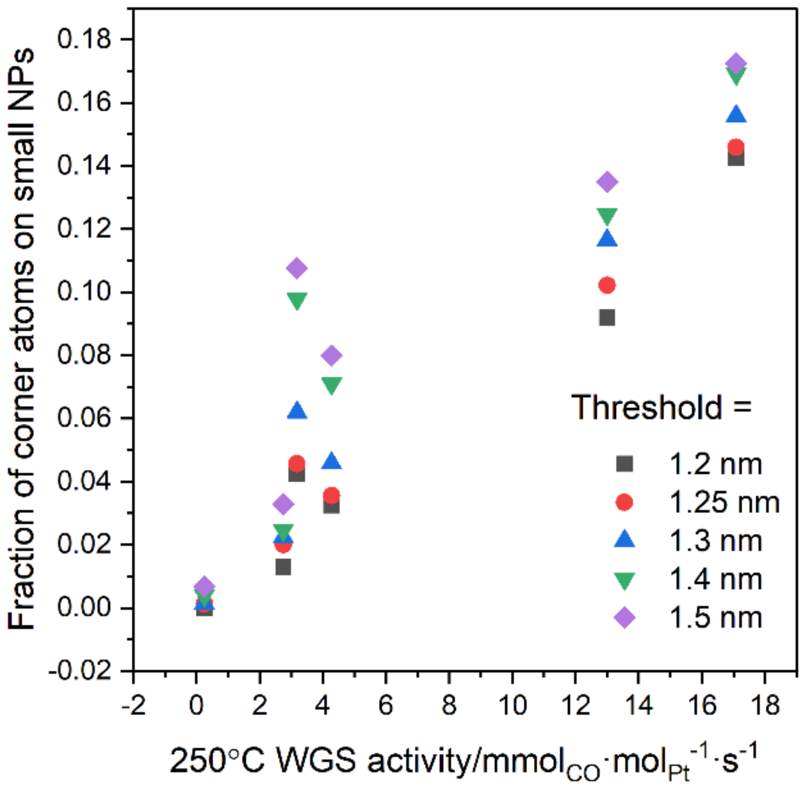

Supplementary Figure 20. Correlation between WGS activity and fraction of corner atoms on small NPs using different threshold (1.2, 1.25, 1.3, 1.4 and 1.5nm) to divide “smaller” and “larger” Pt NPs.

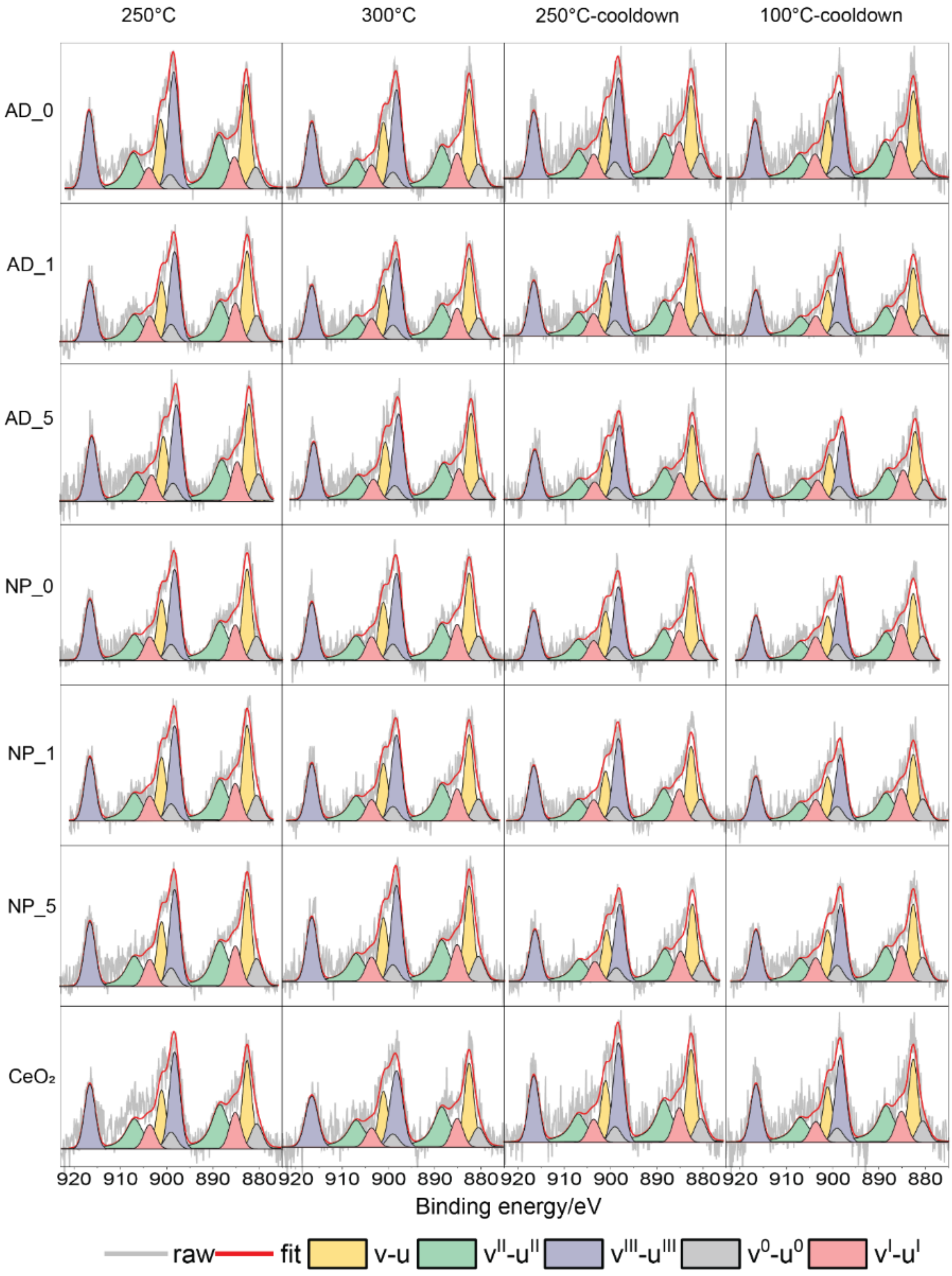

Supplementary Figure 21. Experimental X-ray photoelectron spectra and peak fitting of the *Ce 3d* region over all the samples obtained in 0.1 mbar CO + 0.3 mbar H_2O, during ramp-up at 250-300°C and cool-down at 300-100°C, using 1200 eV of photon energy.

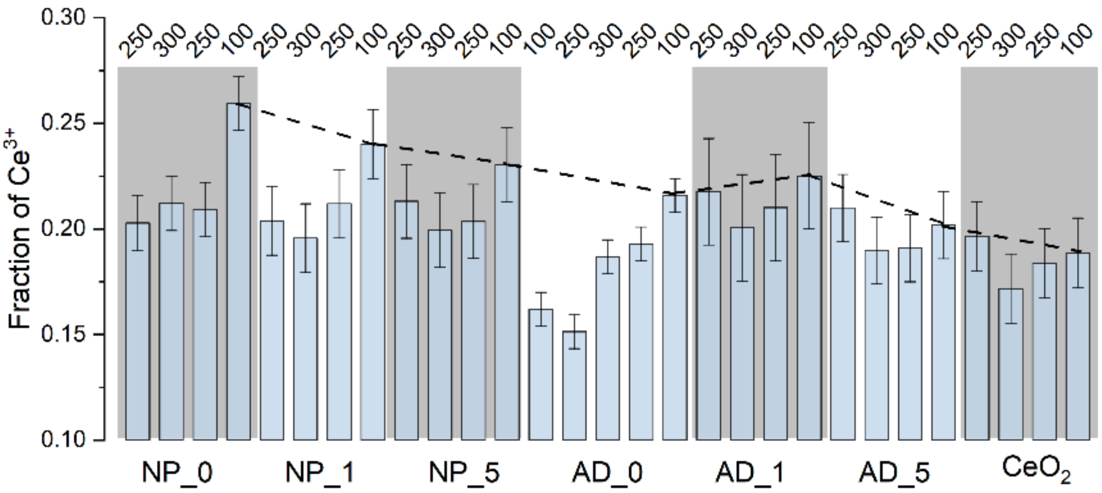

Supplementary Figure 22. Stacked bar plot showing the evolution of *Ce 3d* species on all samples at different temperatures in a 0.1 mbar CO + 0.3 mbar H_2O reaction environment.

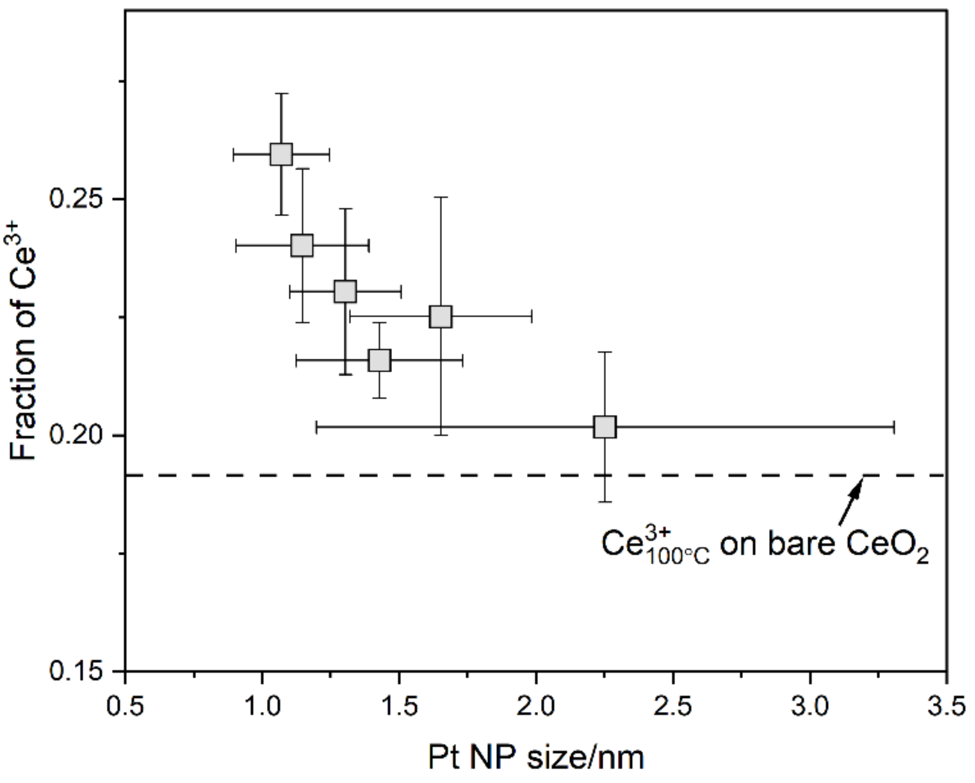

Supplementary Figure 23. Fraction of Ce^{3+} in $CO+H_2O$ at 100°C during cool-down as a function of Pt NP size.

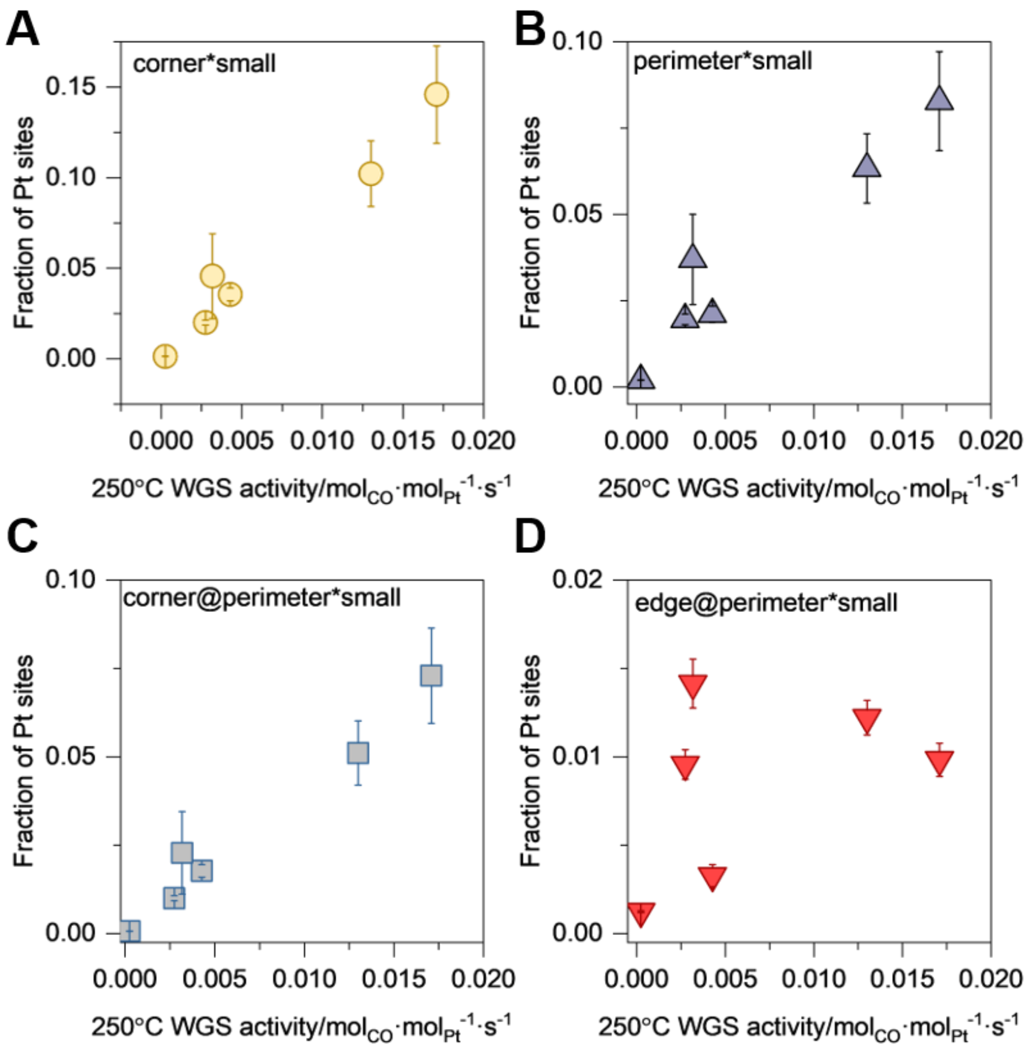

Supplementary Figure 24. Correlation of the fraction of different platinum sites with WGS activity at 250°C.

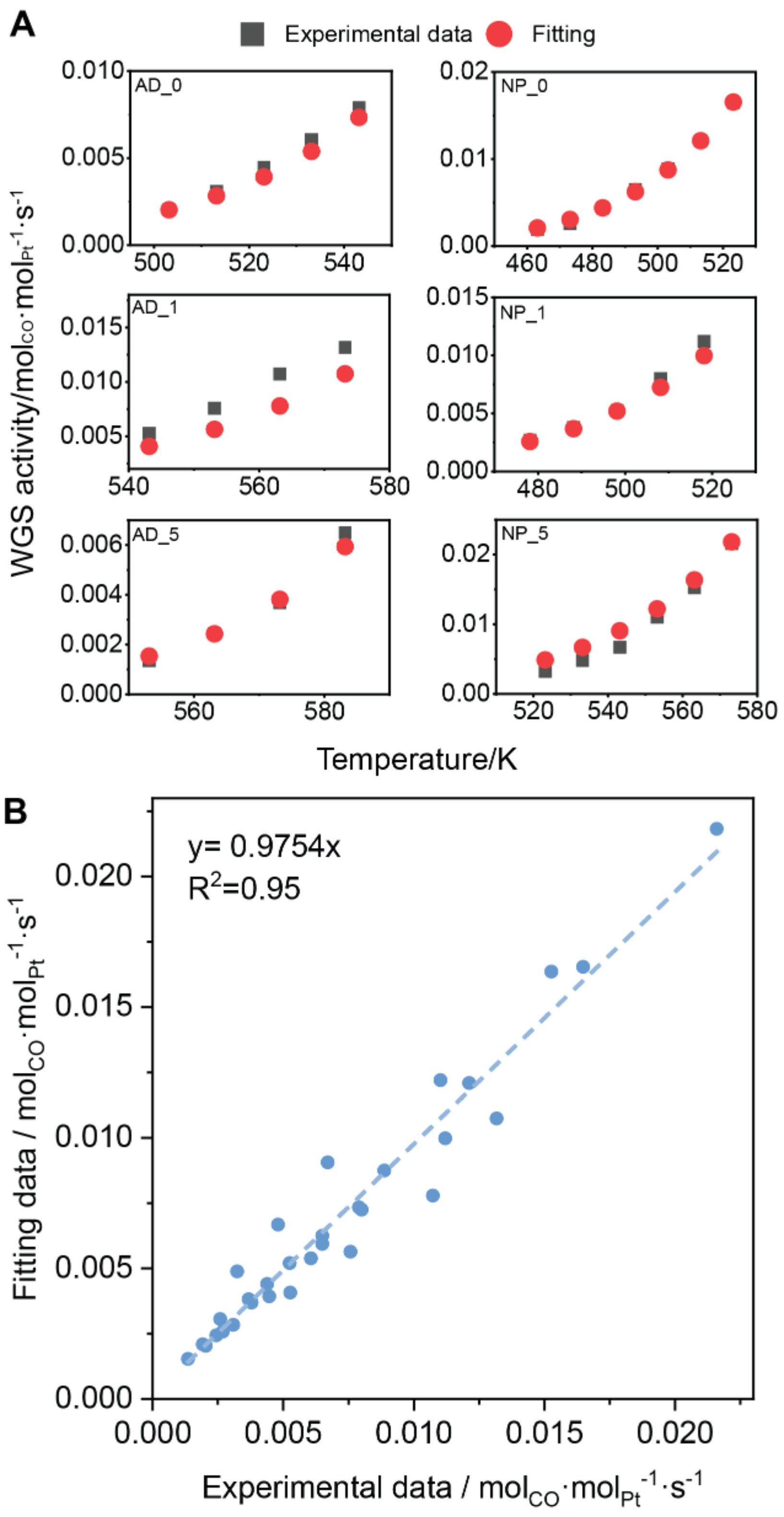

Supplementary Figure 25. (A) Comparison of measured activity data and the fitting by kinetic model as a function of temperature. (B) Overall correlation plot of the fitted data with experimental data.

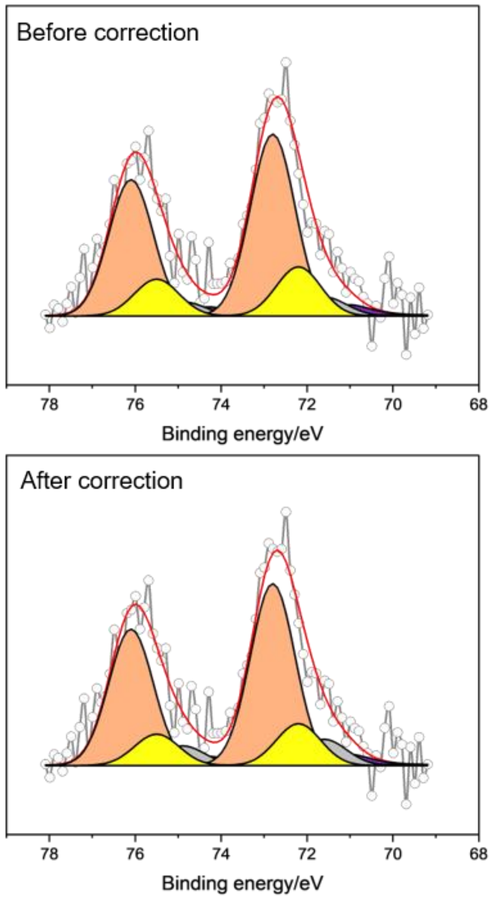

Supplementary Figure 26. Pt 4f peak fitting example of NP_0 sample at 250°C before and after considering electronic structure effect for terrace/corner components.

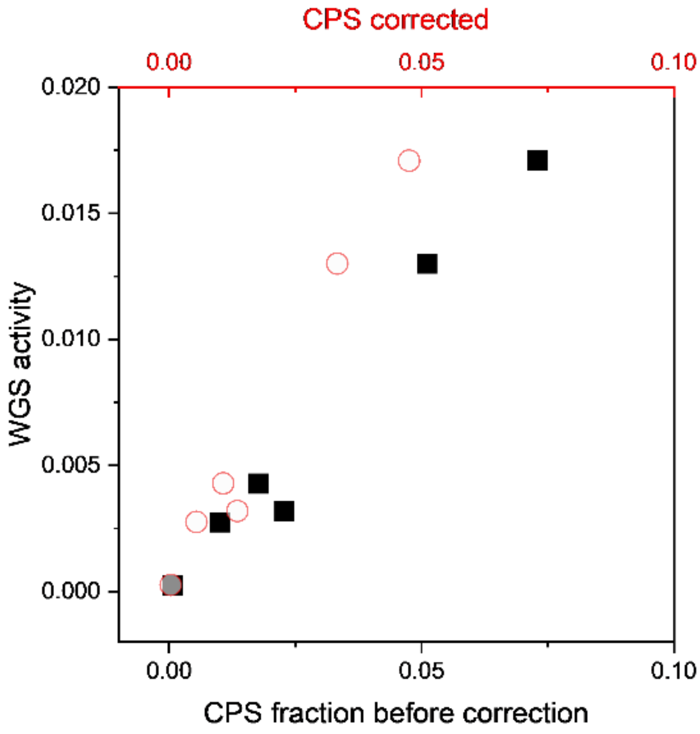

Supplementary Figure 27. Corrections between WGS activity and CPS fraction before and after terrace peak correction considering electronic structure effect.

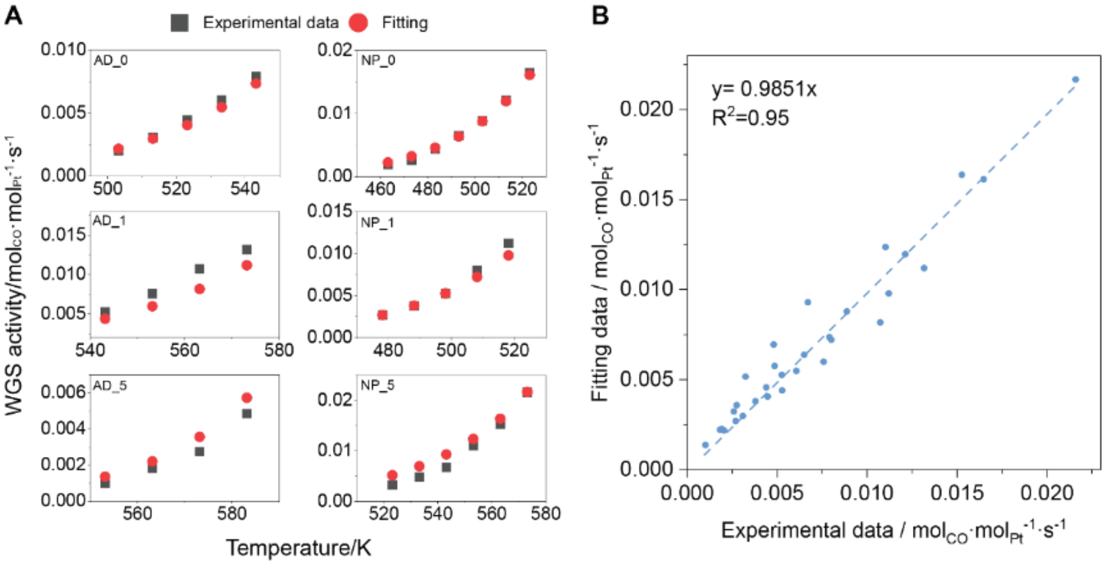

Supplementary Figure 28. Before terrace peak correction considering electronic structure effect (A) Comparison of measured activity data and the fitting by kinetic model as a function of temperature. (B) Correlation of the fitted data and experimental data.

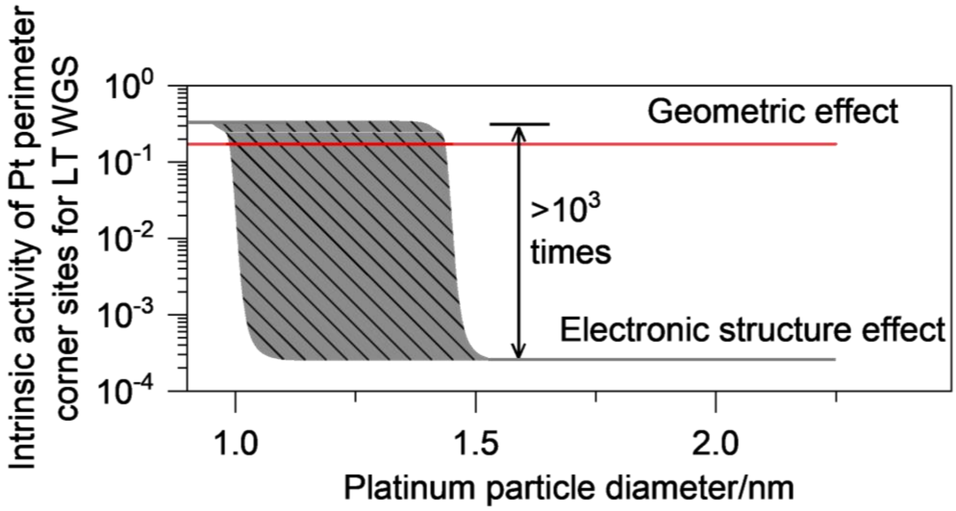

Supplementary Figure 29. Size dependence of intrinsic WGS activity over Pt corner sites at the metal-support interface.

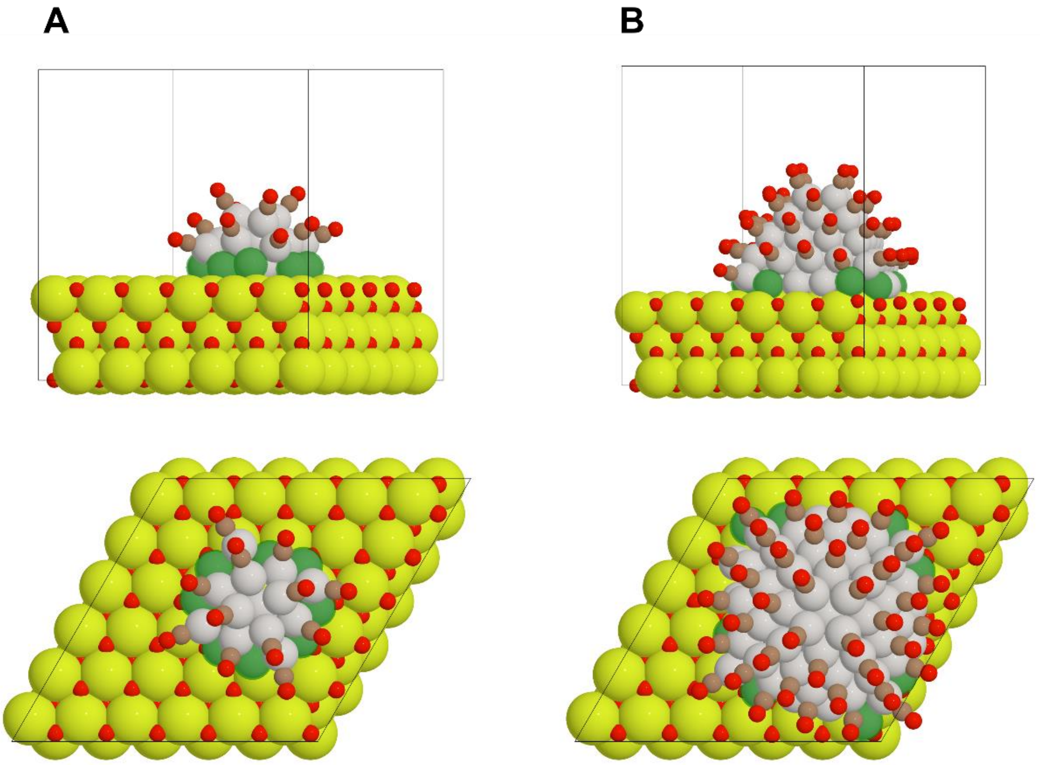

Supplementary Figure 30. The side and top view of the atomic structures of (A) Pt0.9, and (B) Pt1.6, supported on CeO_2(111) . The colors of the Ce, O, Pt and C atoms are yellow, red, light-grey, and dark-grey respectively. Corner Pt atoms are highlighted with green spheres.

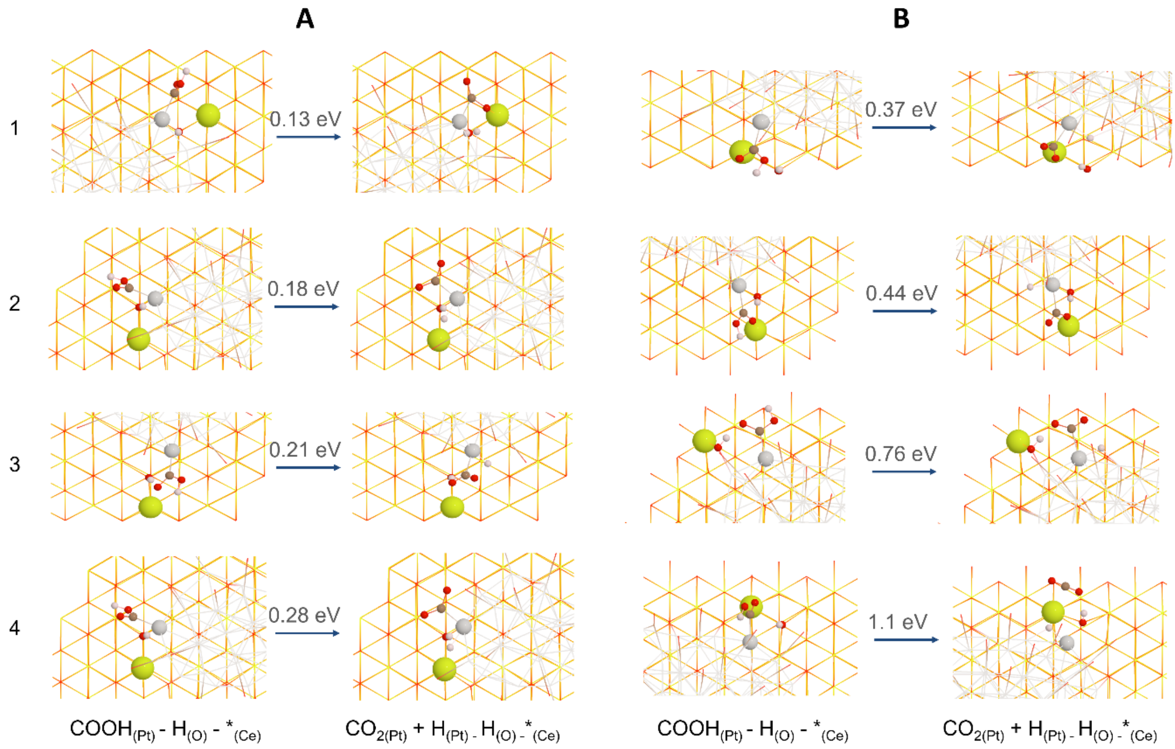

Supplementary Figure 31. The atomic structures of the *COOH dissociation process on (A) Pt0.9 and (B) Pt1.6. Each panel displays key intermediates of the reaction step at the bottom. To maintain the overall stoichiometry of the WGSR, a hydrogen atom is placed on a neighboring lattice oxygen site. The lattice cerium site, where OH was initially adsorbed before forming COOH on the Pt site, is also shown to indicate its origin. In the structure model, only the relevant atoms are highlighted as spheres, and the rest are represented as lines. The colors of the Ce, O, Pt, C and H atoms are yellow, red, light-grey, dark-grey, and white, respectively.

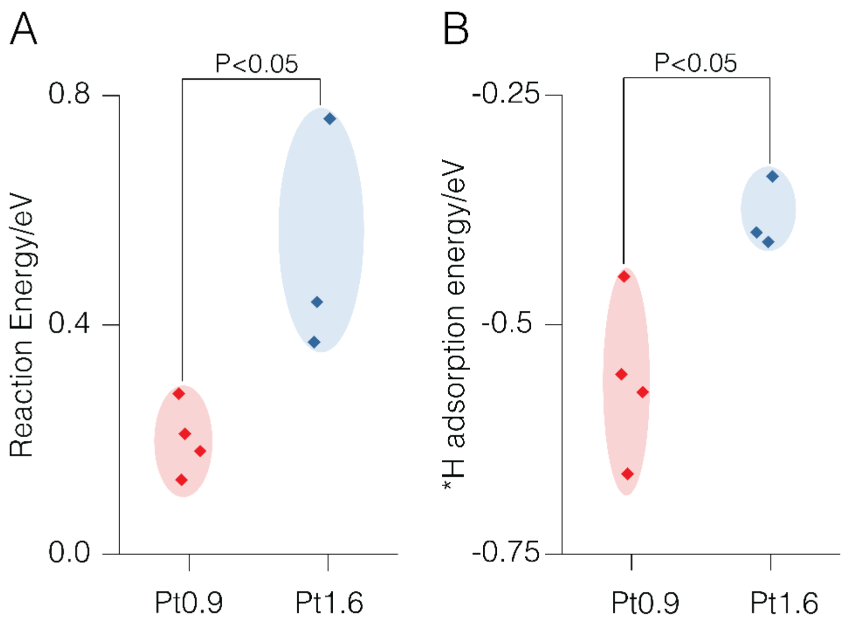

Supplementary Figure 32. Distribution of reaction energies for the carboxyl dissociation and *H adsorption energy after carboxyl dissociation on 0.9 nm (Pt0.9) and 1.6 nm (Pt1.6) Pt NPs on CeO_2.

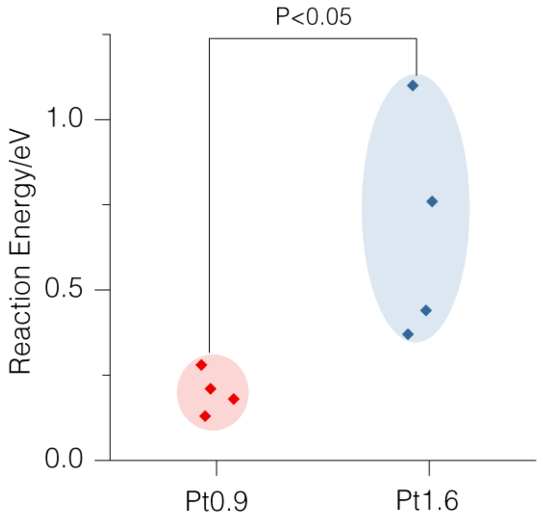

Supplementary Figure 33. Distribution of reaction energies for the carboxyl dissociation on 0.9 nm (Pt0.9) and 1.6 nm (Pt1.6) Pt NPs on CeO_2 when taking the 1.1eV reaction energy into account.

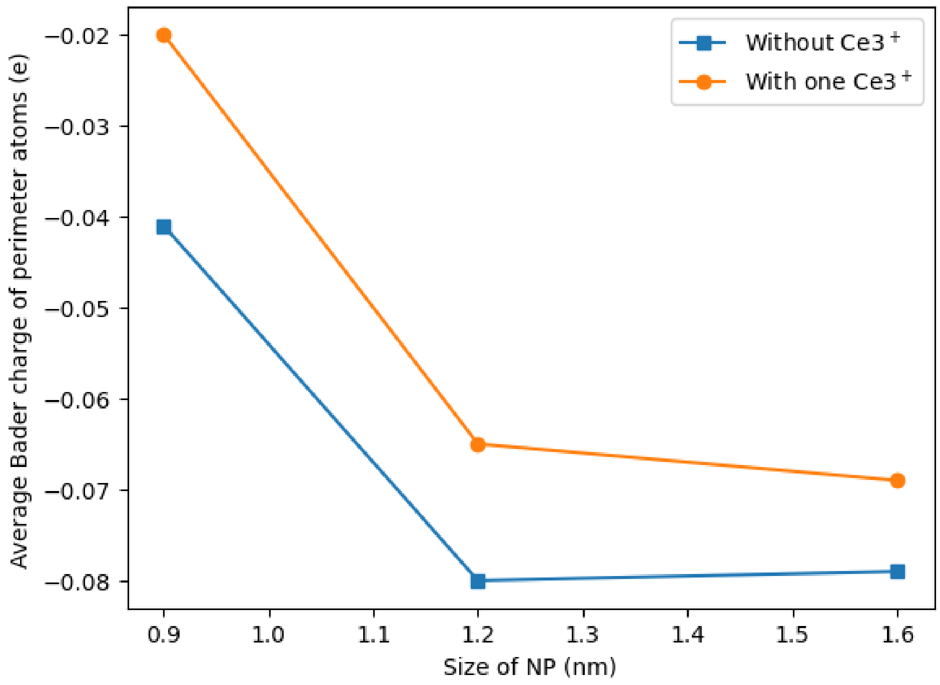

Supplementary Figure 34. Average Bader charge of the perimeter Pt atoms (with and without the formation of one Ce^{3+} cation using the method discussed earlier in the text), highlighting a significant difference of the average change for small nanoparticles.

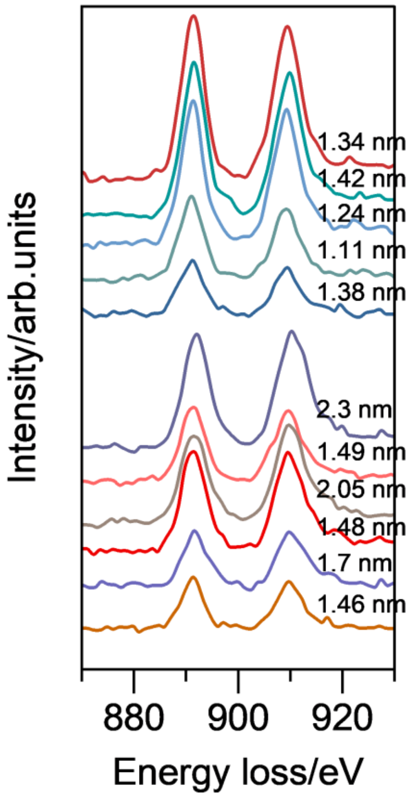

Supplementary Figure 35. Representative Ce $M_{5,4}$ EELS raw spectra of CeO_2 in the vicinity of small and large Pt NPs.

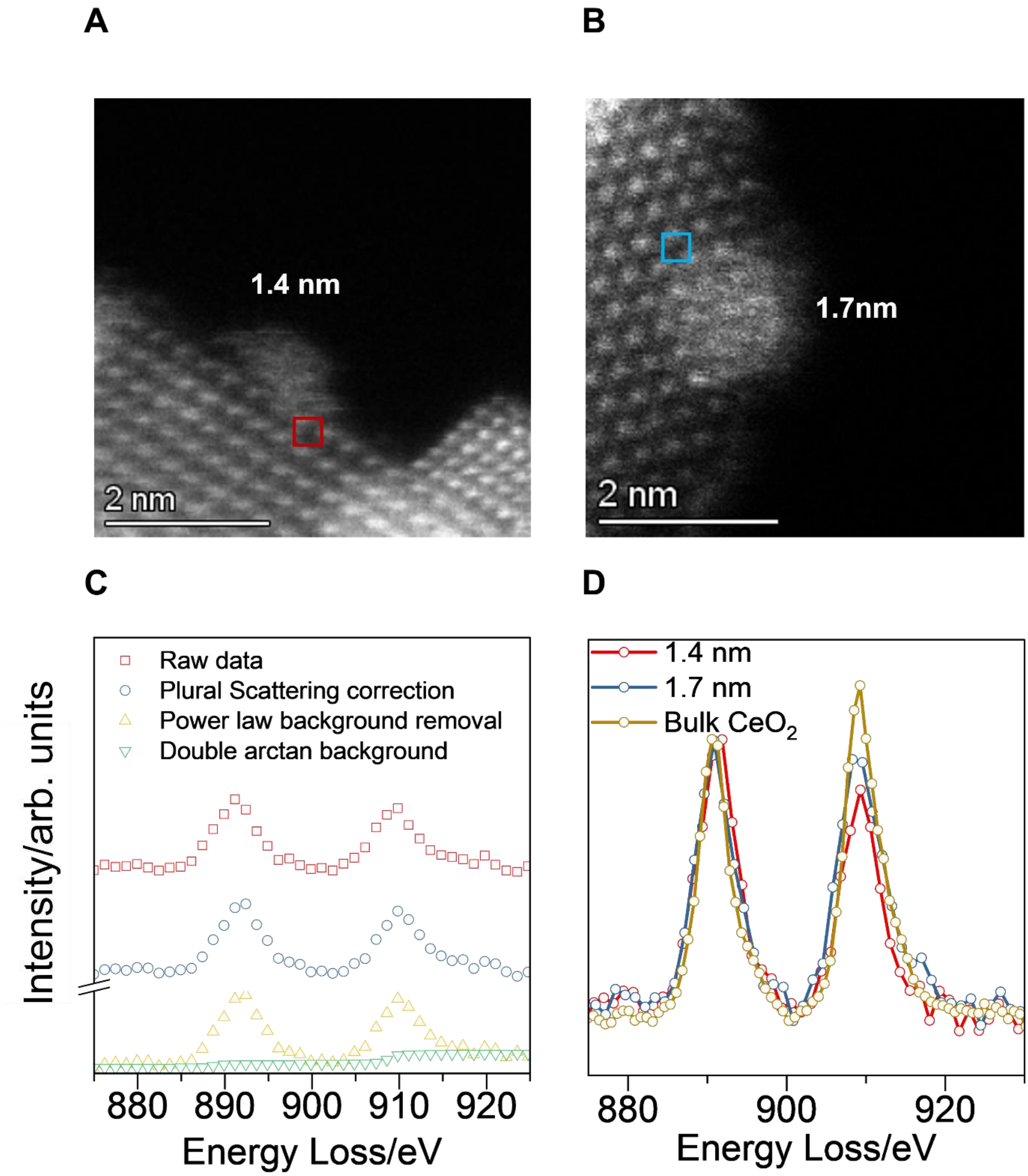

Supplementary Figure 36. HAADF-STEM image of model Pt/CeO_2 catalyst with different size of Pt NPs (A) 1.4 nm and (B) 1.7 nm. The labels indicate the EELS acquisition position. (C) An example of background subtraction for Ce $M_{5,4}$ edge core loss spectrum. (D) Ce $M_{5,4}$ edge EEL spectra of the labelled position on CeO_2 in the vicinity of small and large Pt NPs and bulk CeO_2 after background correction.

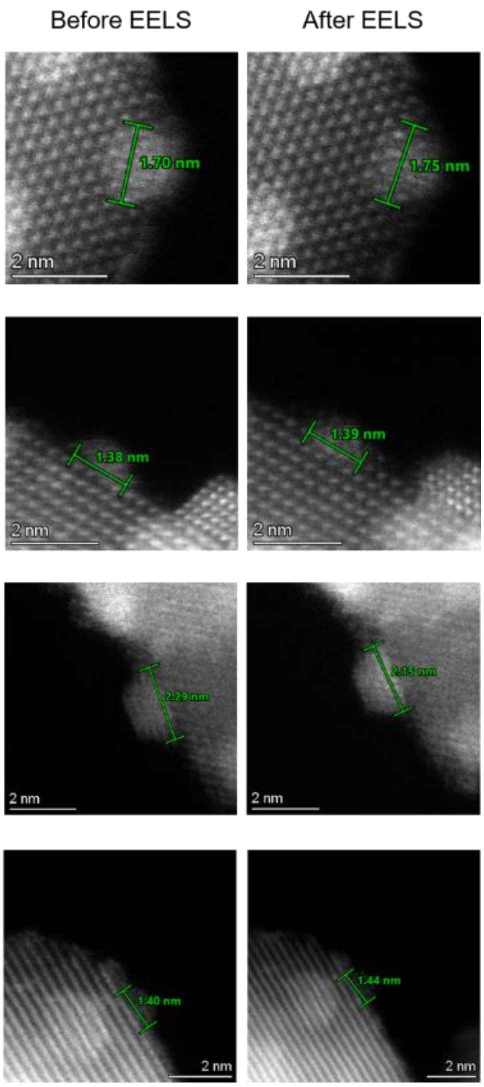

Supplementary Figure 37. Representative HAADF-STEM images of the sample before/after EELS measurement.

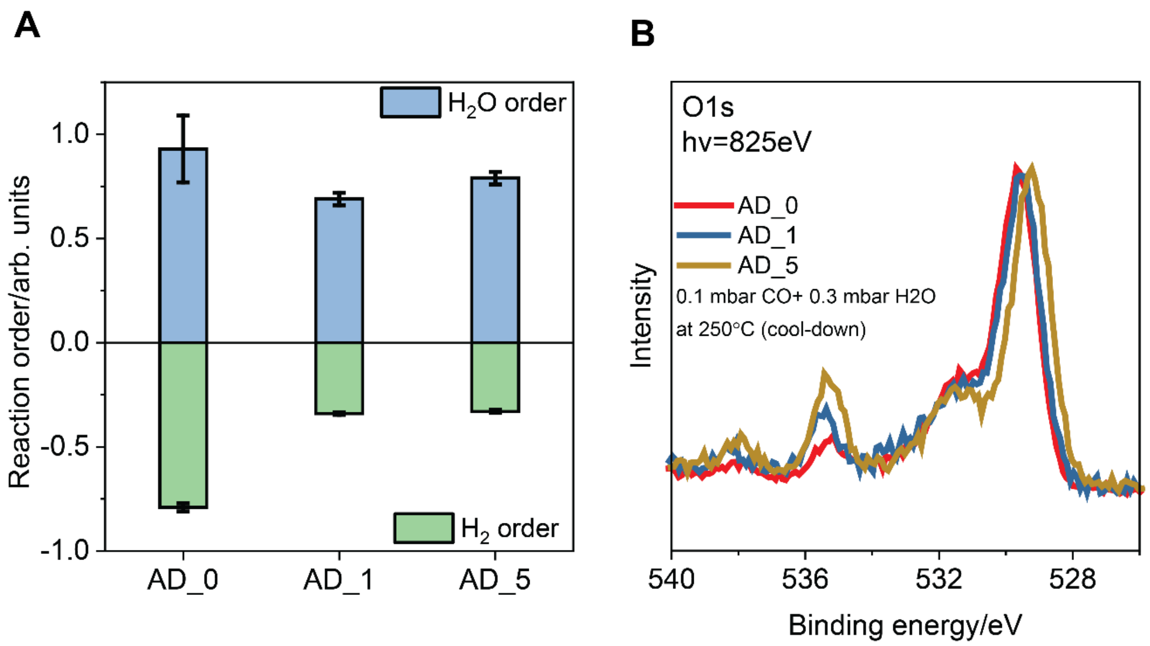

Supplementary Figure 38. (A) Comparison of the reaction orders of H_2 and H_2O for AD_x samples. (B) *O 1s* spectra of AD_x samples in 0.1 mbar CO + 0.3 mbar H_2O at 250 °C during cool-down. The intensity was normalized using lattice oxygen peak intensity.

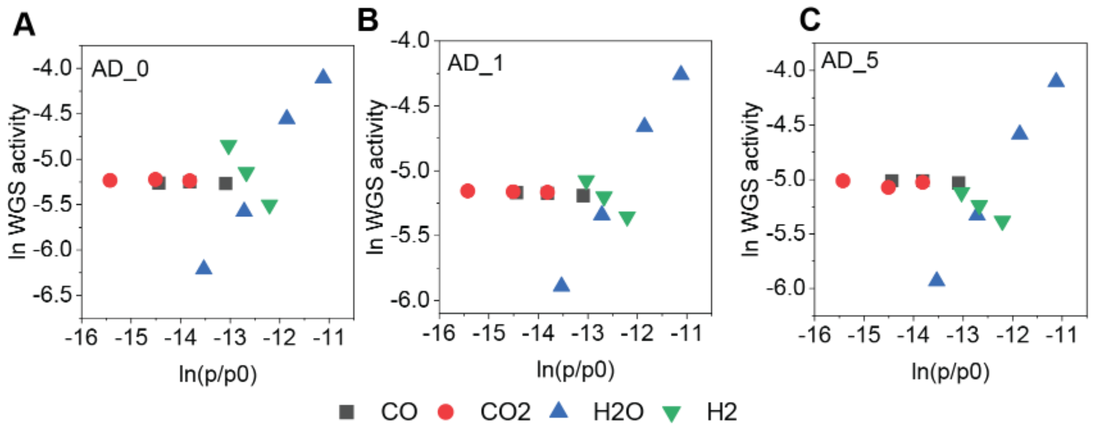

Supplementary Figure 39. Apparent reaction orders for CO, CO_2, H_2, and H_2O for (A) AD_0, (B) AD_1 and (C) AD_5 over WGS reaction. Standard conditions were 0.01% CO, 0.01% CO_2,0.03% H_2O, 0.05% H_2 and balance Ar. Order determination at standard conditions with each component varying in the range of 0.0053 – 0.0206 % for CO, 0.002 – 0.01 % for CO_2, 0.022 – 0.078% for H_2, and 0.013 – 0.148 %for H_2O.

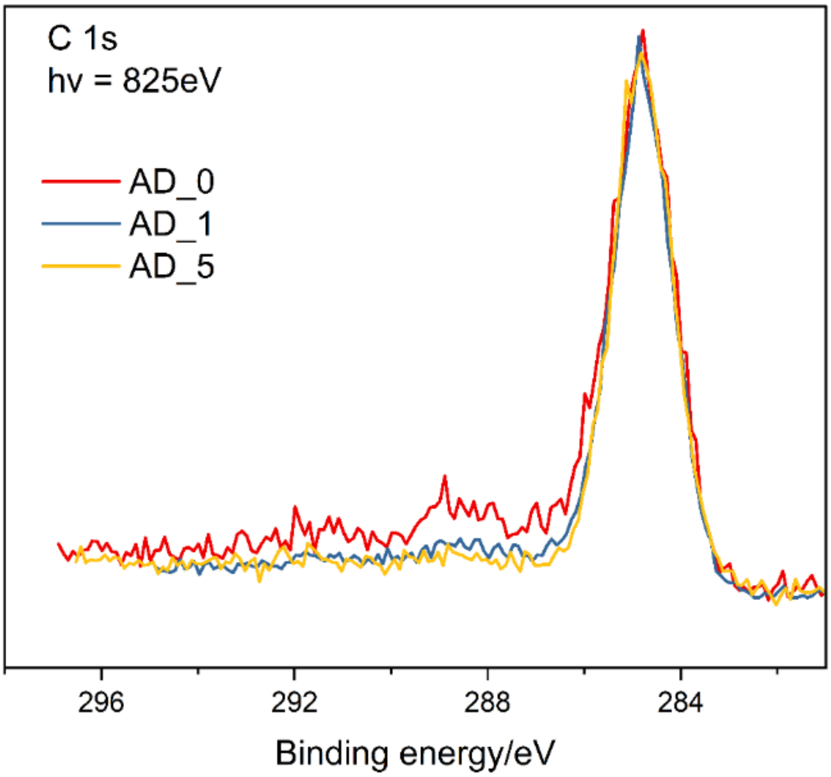

Supplementary Figure 40. *C 1s* spectra of AD_x samples acquired under the same conditions and photon energy with *O 1s* for energy alignment. The intensity was normalized using adventitious carbon peak intensity.

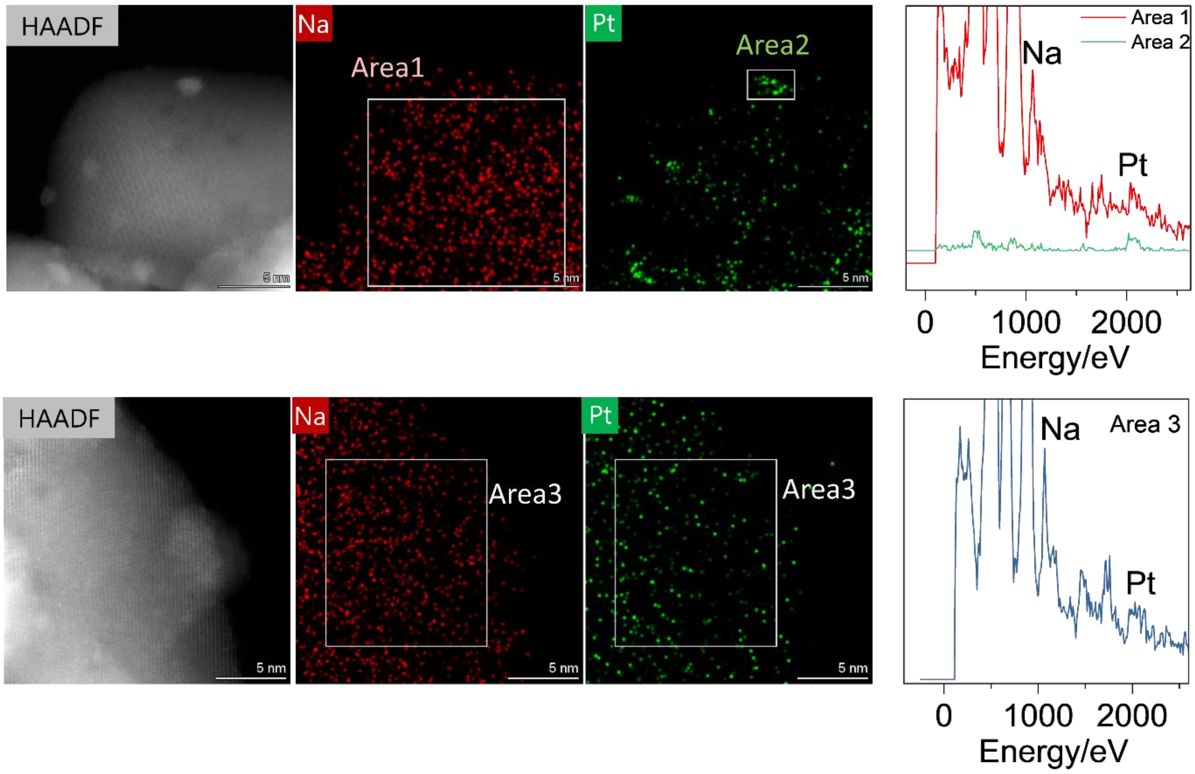

Supplementary Figure 41. STEM and EDXS images of used AD_1. Homogeneous dispersion of sodium is observed in two representative regions containing Pt NPs and Pt ADs.

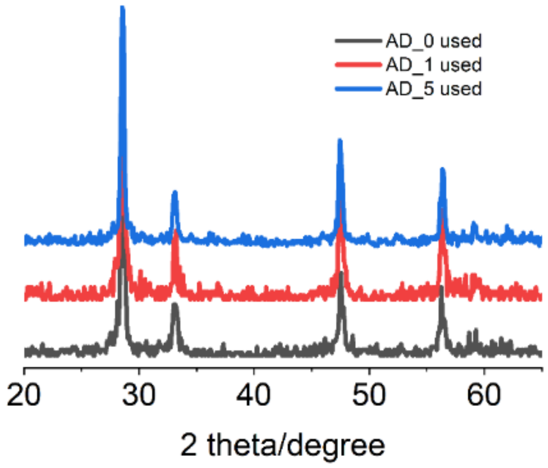

Supplementary Figure 42. *ex-situ* XRD patterns of used AD_x samples after WGS in air at RT.

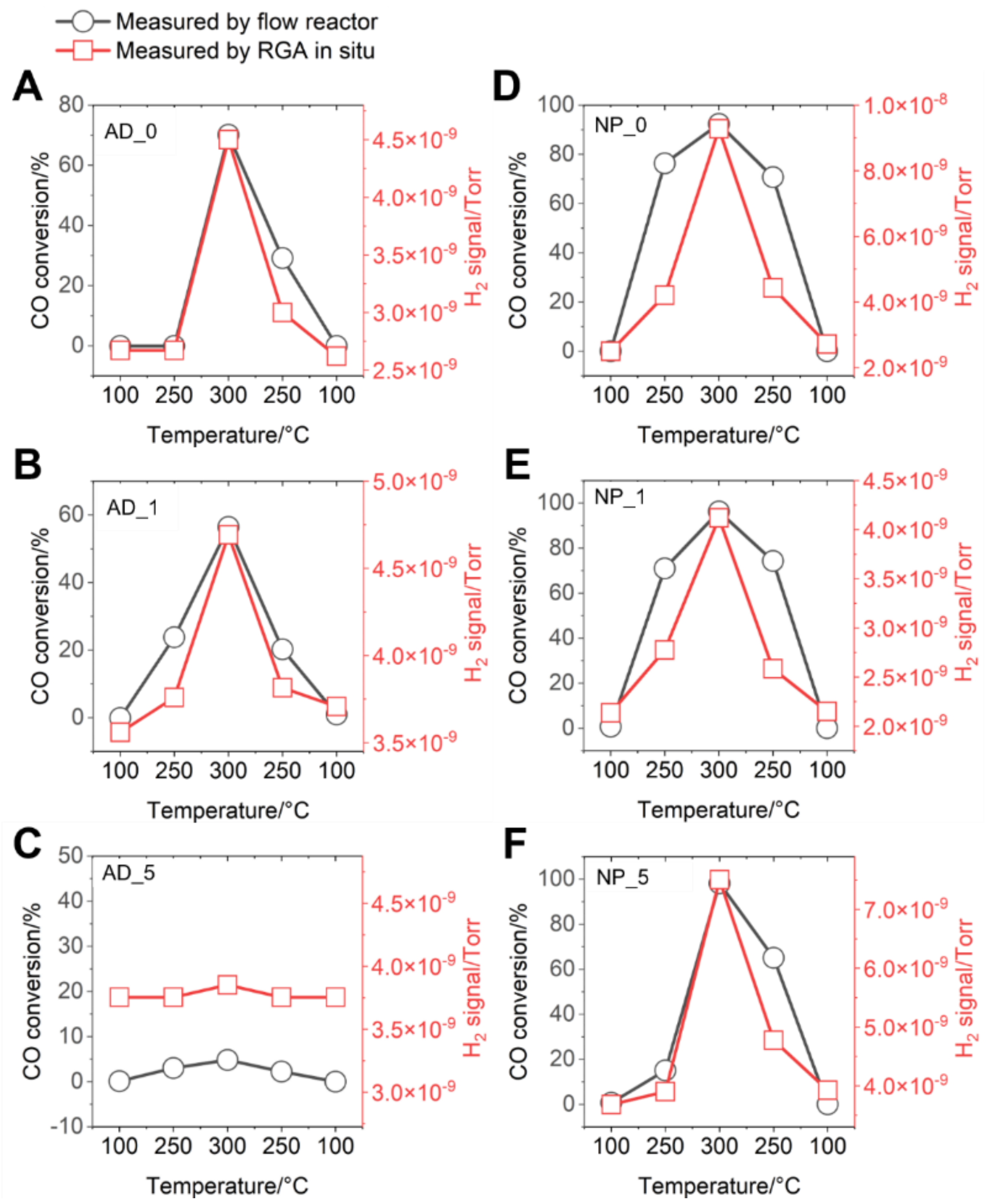

Supplementary Figure 43. WGS performance of all the samples measured during APXPS by mass spectrometer in terms of H_2 production and the CO conversion in flow reactors when fast ramp-up and cool-down at 100-300-100°C in 100 ppm CO + 300 ppm H_2O.

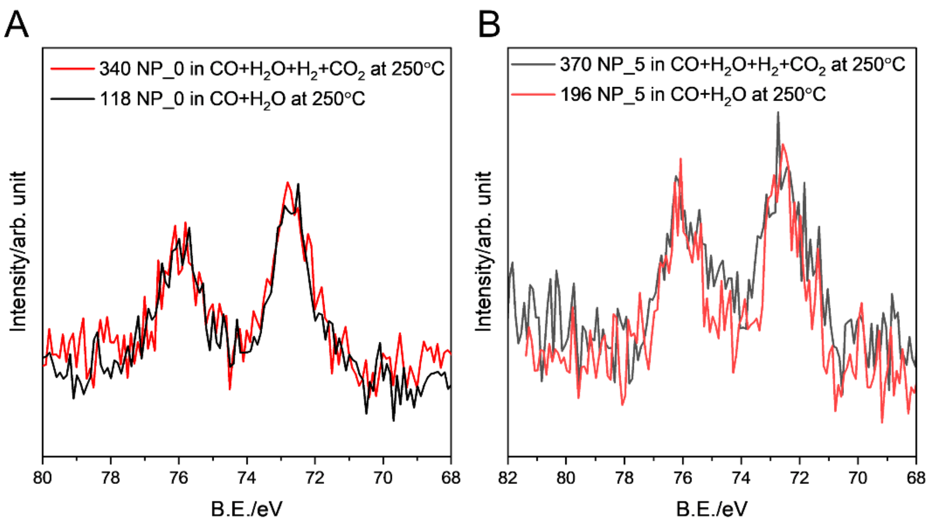

Supplementary Figure 44. Comparison of raw *Pt 4f* XP spectra collected at 250ºC in (**A**) CO+H_2O (0.1+0.3 mbar) and (**B**) CO+H_2O+CO_2+H_2 (0.1+0.3+0.1+0.5 mbar) for NP_0 and NP_5.

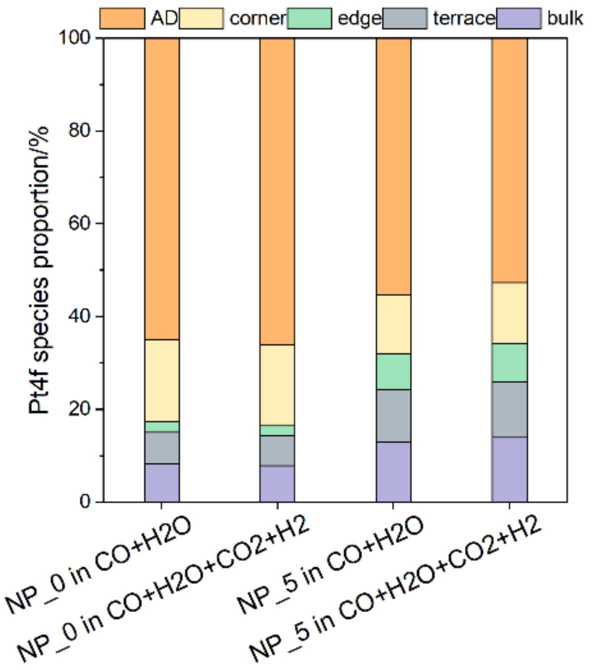

Supplementary Figure 45. Comparison of *Pt 4f* species proportion in XP spectra collected in $CO+H_2O$ and $CO+H_2O+CO_2+H_2$ for NP_0 and NP_5.

Supplementary Table 1. Sample names and preparation methods in this work.

Sample name	Pt loading/ wt%	Platinum precursor	Atomic ratio of Na: Pt	Surface Pt species in STEM
AD_0	1	H_2PtCl_6	0	AD
AD_1	1	H_2PtCl_6	1	AD
AD_5	1	H_2PtCl_6	5	AD
NP_0	1	$Pt(NH_3)_4](NO_3)_2$	0	NP+AD
NP_1	1	$Pt(NH_3)_4](NO_3)_2$	1	AD
NP_5	1	$Pt(NH_3)_4](NO_3)_2$	5	AD

Supplementary Table 2. Platinum particle diameters(nm) of used samples as determined from STEM images.

AD_0-used	AD_1-used	AD_5-used	NP_0-used	NP_1-used	NP_5-used
2.18	1.68	1.78	0.89	0.96	1.29
2.03	2.08	1.68	0.92	0.91	1.35
1.68	1.39	1.98	0.99	0.91	1.39
1.58	1.44	2.23	0.79	1.14	1.25
1.83	2.62	1.88	1.22	0.88	1.39
2.33	2.23	2.18	0.96	1.10	1.06
1.49	1.19	1.73	1.09	1.58	1.06
1.29	1.24	2.33	0.96	1.06	0.92
1.54	1.78	1.88	0.99	0.99	1.06
1.58	1.58	1.58	1.06	1.02	1.49
1.34	1.98	1.78	1.06	1.06	1.39
1.29	1.54	1.68	1.12	1.19	1.06
1.14	2.03	1.39	0.83	1.19	1.03
1.68	1.78	1.78	1.35	1.29	1.49
1.58	1.68	1.63	1.06	1.22	1.34
1.78	1.88	1.83	1.06	0.72	1.75
1.39	1.83	1.88	1.13	1.49	1.29
1.63	1.58	1.93	0.79	1.04	1.32
1.19	1.78	1.49	0.77	1.14	1.25
1.19	1.83	1.78	0.85	0.89	1.62
1.58	1.58	1.98	1.11	1.02	1.32
1.09	1.68	1.88	0.86	0.89	1.39
1.29	1.14	2.13	1.06	0.83	1.09
1.39	2.08	1.98	0.89	1.12	1.32
1.63	1.58	1.63	0.96	1.27	1.42
0.84	1.78	2.53	1.02	1.11	1.12
1.19	1.93	1.39	0.99	0.89	1.39
1.39	1.78	1.68	1.12	1.46	1.63
2.28	2.33	2.33	1.45	1.26	1.54
1.63	1.83	2.13	1.19	1.78	1.19
1.39	1.83	1.44	1.31	0.69	1.25
1.68	2.15	2.62	1.19	1.04	1.32
1.34	1.58	3.37	1.12	1.14	1.06
1.45	1.75	2.38	0.79	1.29	1.22
1.32	1.49	2.23	1.06	1.19	1.19
1.22	1.52	5.41	1.06	1.39	1.19
1.88	1.54	4.56	1.52	1.19	1.68
1.73	1.88	6.40	1.35	0.84	1.68
1.54	1.98	1.85	1.25	1.29	1.11
1.09	1.63	1.68	0.99	1.34	
1.39	2.48	1.55	1.29	1.09	
0.89	1.09	1.73	1.09	1.14	
1.04	1.24	1.63	1.12	1.49	
1.93	1.19	2.67	1.12	1.24	
1.83	1.24	1.24	1.09	0.99	
1.14	1.24	1.83	1.19	0.87	
1.78	1.98	1.68	1.29	0.99	
1.19	1.83	1.58	1.09	1.09	
1.19	2.18	4.65	1.39	0.67	
1.09	1.29	2.38	1.02	0.99	
1.09	1.58	4.46	1.12	0.89	
1.14	1.63	3.76	0.86	1.22	
1.39	1.78	2.18	0.89	1.25	
1.39	1.44			1.09	
1.14	1.54			1.19	

1.58	1.58			0.63	
1.58	1.44			1.25	
1.34	1.24			1.24	
1.98	1.54			1.44	
1.54	1.98			1.14	
1.73	1.34			1.14	
1.39	1.73			0.99	
1.39	2.18			1.34	
1.68	2.08			1.63	
1.39	1.68			1.78	
1.44	1.93			1.24	
1.34	1.78			1.49	
1.34	1.63			1.19	
1.58	2.03			1.56	
1.44	1.19			1.34	
1.14	1.78			1.04	
1.29	1.78				
1.29	1.49				
1.24	1.78				
1.45	1.88				
1.25	1.83				
1.62	1.78				
0.92	1.58				
0.89	1.25				
1.25	1.16				
1.19	1.68				
1.45	1.85				
1.12	1.19				
0.96	1.58				
1.39	1.42				
	1.19				
	1.42				
	1.22				
	1.55				
	1.19				
	1.02				
	1.12				

Supplementary Table 3. Peak Fitting Parameters (Position, FWHM, Lorentzian/Gaussian ratio and Asymmetry parameters) of *Pt 4f* Photoemission Spectra.

Pt species	Position/eV	FWHM/eV	Lorentzian/Gaussian ratio	Asymmetry parameters	
				TS	TL
Bulk Pt^0	71.0±0.05	1.22±0.05	80	0.3	110
Terrace Pt^0	71.6±0.05	1.22±0.05	0	0	1
Edge Pt^0	72.2±0.05	1.22±0.05	0	0	1
Corner Pt^0	72.2±0.05	1.22±0.05	0	0	1
AD Pt^{2+}	72.8±0.1	1.22±0.05	0	0	1

Supplementary Table 4. Number of atoms at different sites of NP models in the 0.88-2.38 nm size.

d_{NP}(nm)	Number of atoms	Bulk atoms	Terrace atoms	Edge atoms	Corner atoms
0.88	25	5	4	0	16
1.24	50	14	12	8	16
1.62	86	30	24	16	16
2	135	55	40	24	16
2.38	199	91	60	32	16

Supplementary Table 5. Fitting parameters used for *Pt 4f* photoemission spectra peak fitting.

Sample	d_{NP}(nm)	Bulk/terrace ratio	Terrace/ terrace ratio	Edge/ terrace ratio	Corner/ terrace ratio
AD_0	1.43	1.206	1	0.671	1
AD_1	1.65	1.259	1	0.654	0.654
AD_5	2.25	1.473	1	0.555	0.302
NP_0	1.07	1.206	1	0.348	2.582
NP_1	1.15	1.183	1	0.478	2
NP_5	1.30	1.171	1	0.708	1.091

Supplementary Table 6. Error estimation of measurement and peak fitting for Pt 4f spectra in this work.

**Sample**	**AD_0**					**AD_1**				**AD_5**			
Temperature/C	100	250	300	250	100	250	300	250	100	250	300	250	100
fitting error	0.029	0.033	0.033	0.035	0.047	0.024	0.030	0.035	0.033	0.026	0.031	0.038	0.034
measure error bulk	0	0	0.008	0.008	0.008	0.016	0.016	0.016	0.016	0.001	0.001	0.001	0.001
fit error bulk	0	0	0.002	0.001	0.003	0.004	0.007	0.007	0.007	0.008	0.011	0.013	0.012
error bulk	0	0	0.008	0.008	0.008	0.017	0.017	0.018	0.018	0.008	0.011	0.013	0.012
measure error terrace	0	0	0.007	0.007	0.007	0.013	0.013	0.013	0.013	0.001	0.001	0.001	0.001
fit error terrace	0	0	0.002	0.001	0.002	0.003	0.005	0.006	0.006	0.006	0.007	0.009	0.008
error terrace	0	0	0.007	0.007	0.007	0.013	0.014	0.014	0.014	0.006	0.007	0.009	0.008
measure error edge	0	0	0.004	0.004	0.004	0.008	0.008	0.008	0.008	0.000	0.000	0.000	0.000
fit error edge	0	0	0.001	0.001	0.002	0.002	0.004	0.004	0.004	0.003	0.004	0.005	0.004
error edge	0	0	0.005	0.004	0.005	0.009	0.009	0.009	0.009	0.003	0.004	0.005	0.004
measure error corner	0	0	0.012	0.012	0.012	0.006	0.006	0.006	0.006	0.000	0.000	0.000	0.000
fit error corner	0	0	0.003	0.004	0.005	0.004	0.004	0.004	0.004	0.002	0.002	0.003	0.002
error corner	0	0	0.012	0.013	0.013	0.007	0.007	0.007	0.007	0.002	0.002	0.003	0.002
measure error AD	0	0	0.013	0.013	0.013	0.049	0.049	0.049	0.049	0.002	0.002	0.002	0.002
fit error AD	0.029	0.033	0.025	0.027	0.034	0.010	0.011	0.015	0.013	0.008	0.007	0.008	0.007
error AD	0.029	0.033	0.028	0.030	0.037	0.050	0.050	0.051	0.051	0.008	0.007	0.008	0.007
**sample**	**NP_0**				**NP_1**				**NP_5**				
Temperature/C	250	300	250	100	250	300	250	100	250	300	250	100	
Fitting error	0.020	0.018	0.029	0.026	0.027	0.027	0.027	0.032	0.031	0.183	0.034	0.029	
measure error bulk	0.008	0.008	0.008	0.008	0.006	0.006	0.006	0.006	0.012	0.012	0.012	0.012	
fit error bulk	0.001	0.002	0.002	0.002	0.002	0.002	0.002	0.002	0.003	0.019	0.004	0.004	
error bulk	0.008	0.008	0.008	0.008	0.007	0.007	0.007	0.007	0.012	0.022	0.013	0.013	
measure error terrace	0.006	0.006	0.006	0.006	0.005	0.005	0.005	0.005	0.010	0.010	0.010	0.010	
fit error terrace	0.001	0.002	0.002	0.002	0.002	0.002	0.002	0.002	0.002	0.016	0.004	0.004	
error terrace	0.006	0.006	0.007	0.006	0.006	0.006	0.006	0.006	0.010	0.019	0.011	0.011	
measure error edge	0.002	0.002	0.002	0.002	0.003	0.003	0.003	0.003	0.007	0.007	0.007	0.007	
fit error edge	0.000	0.001	0.001	0.001	0.001	0.001	0.001	0.001	0.002	0.011	0.003	0.003	
error edge	0.002	0.002	0.002	0.002	0.003	0.003	0.003	0.003	0.007	0.013	0.008	0.008	
measure error corner	0.032	0.032	0.032	0.032	0.025	0.025	0.025	0.025	0.065	0.065	0.065	0.065	
fit error corner	0.003	0.004	0.005	0.004	0.004	0.004	0.004	0.004	0.002	0.030	0.004	0.004	
error corner	0.032	0.032	0.032	0.032	0.026	0.026	0.026	0.026	0.065	0.072	0.065	0.065	
measure error AD	0.016	0.016	0.016	0.016	0.011	0.011	0.011	0.011	0.014	0.014	0.014	0.014	
fit error AD	0.015	0.010	0.019	0.018	0.018	0.018	0.018	0.022	0.023	0.107	0.019	0.014	
error AD	0.022	0.019	0.025	0.024	0.021	0.021	0.021	0.025	0.027	0.108	0.024	0.020	

Supplementary Table 7. Number and proportion of platinum particles smaller than 1.25 nm in the STEM images and their average diameters together with the corresponding proportion of terrace and corner atoms on small NPs.

Sample name	Number of particles smaller than 1.25 nm	Proportion of particles smaller than 1.25 nm(%)	Average diameter of particles smaller than 1.25nm (nm)	Proportion of terrace atoms in particles smaller than 1.25 nm(%)	Proportion of corner atoms in particles smaller than 1.25 nm(%)
AD_0	24	28	1.11	27.4	42.0
AD_1	17	18	1.19	29.6	35.0
AD_5	1	1.9	1.24	30.1	32.8
NP_0	44	83	1.02	25.7	48.2
NP_1	50	70	1.04	25.7	48.2
NP_5	14	36	1.12	27.6	40.2

Supplementary Table 8. Multi-linear regression results of WGS rate with bulk, terrace, edge and corner sites on small NPs (<1.25 nm).

Site	Coefficient*	R^2
Bulk	0	0.974
Terrace	0	
Edge	0	
Corner	0.1204	

*sklearn.linear_model.LinearRegression[65] in Python was employed to fit the raw data. Parameter "positive=True" was applied to constrain the coefficients no smaller than 0.

Supplementary Table 9. Peak Fitting Parameters (Position, FWHM, Lorentzian/Gaussian ratio and Asymmetry parameters) of *Ce 3d* Photoemission Spectra.

Ce 3d peak	Position/eV	FWHM/eV	Lorentzian/Gaussian ratio	Area constraints	
				v^i/v^0	v^i/v
v^0	880.56±0.05	3.0±0.05	0	1	-
v^I	885.16±0.05	3.0±0.05	0	1.46	-
v	882.6±0.05	2.4±0.05	0	-	1
v^{II}	888.5±0.05	4.3±0.05	90±10	-	1.11
v^{III}	898.3±0.05	2.7±0.05	0	-	1.13

Supplementary Table 10. Experimental data used for the kinetic model fitting

T in K	r_{eff}	*x(CPS fraction)*	*1-x*	*sample name*
503.15	0.002036934	0.01074	0.98926	AD_0
513.15	0.003088254	0.01074	0.98926	AD_0
523.15	0.004468112	0.01074	0.98926	AD_0
533.15	0.00606152	0.01074	0.98926	AD_0
543.15	0.007901331	0.01074	0.98926	AD_0
543.15	0.005269697	0.00542	0.99458	AD_1
553.15	0.007574491	0.00542	0.99458	AD_1
563.15	0.010729596	0.00542	0.99458	AD_1
573.15	0.013168649	0.00542	0.99458	AD_1
553.15	0.00135777	0.000353	0.9996	AD_5
563.15	0.002441097	0.000353	0.9996	AD_5
573.15	0.003683312	0.000353	0.9996	AD_5
583.15	0.006499962	0.000353	0.9996	AD_5
463.15	0.001921033	0.0476	0.9524	NP_0
473.15	0.002589466	0.0476	0.9524	NP_0
483.15	0.004389794	0.0476	0.9524	NP_0
493.15	0.006498717	0.0476	0.9524	NP_0
503.15	0.008871824	0.0476	0.9524	NP_0
513.15	0.012119474	0.0476	0.9524	NP_0
523.15	0.016481939	0.0476	0.9524	NP_0
478.15	0.002689474	0.03334	0.96666	NP_1
488.15	0.003788387	0.03334	0.96666	NP_1
498.15	0.005253229	0.03334	0.96666	NP_1
508.15	0.008005562	0.03334	0.96666	NP_1
518.15	0.011206889	0.03334	0.96666	NP_1
523.15	0.003241946	0.01354	0.98646	NP_5
533.15	0.004807024	0.01354	0.98646	NP_5
543.15	0.006707476	0.01354	0.98646	NP_5
553.15	0.011022618	0.01354	0.98646	NP_5
563.15	0.015270686	0.01354	0.98646	NP_5
573.15	0.021598071	0.01354	0.98646	NP_5

Supplementary Table 11. Fitting results using ODR function in OriginPro and Curve_fit function in Python.

Method	*Results fitted by ODR in OriginPro*	*Results fitted by Curve_fit in Python*
E_1/J·mol^{-1}	68919.00354	69072.97
E_2/J·mol^{-1}	129792.0744	129547.27
A_1/ mol_{CO}·mol_{Pt}^{-1}·s^{-1}	2624410.781	2701723.16
A_2/ mol_{CO}·mol_{Pt}^{-1}·s^{-1}	2270780000	2138681180.00
r_1 at 250°C / mol_{CO}·mol_{CPS}^{-1}·s^{-1}	0.344722342	0.342535102
r_2 at 250°C/ mol_{CO}·mol_{other}^{-1}·s^{-1}	0.000249136	0.000248228
r_1/r_2 at 250 °C	1384	1380
r_1 at 300°C/ mol_{CO}·mol_{CPS}^{-1}·s^{-1}	1.373401925	1.368908544
r_2 at 300°C/ mol_{CO}·mol_{other}^{-1}·s^{-1}	0.003365177	0.003336491
r_1/r_2 at 300 °C	408	410

Supplementary Table 12. Estimation of E_a using the fitted results at the Arrhenius measurement

Sample name	*AD_0*	*AD_1*	*AD_5*	*NP_0*	*NP_1*	*NP_5*
Temperature for estimation/°C	250	285	295	220	225	275
Calculated r_1 by ODR / $mol_{CO}\cdot mol_{CPS}^{-1}\cdot s^{-1}$	0.3447	0.9311	1.2093	0.1315	0.1556	0.7101
Calculated r_2 by ODR / $mol_{CO}\cdot mol_{other}^{-1}\cdot s^{-1}$	0.00025	0.00162	0.00265	0.00004	0.00006	0.00097
Calculated E_a by ODR /kJ·mol^{-1}	72.72	83.64	121.34	69.29	69.54	74.44
Calculated r_1 by Curve_fit / $mol_{CO}\cdot mol_{CPS}^{-1}\cdot s^{-1}$	0.3425	0.9272	1.2050	0.1304	0.1544	0.7067
Calculated r_2 by Curve_fit / $mol_{CO}\cdot mol_{other}^{-1}\cdot s^{-1}$	0.00025	0.00161	0.00263	0.00004	0.00006	0.00097
Calculated E_a by Curve_fit /kJ·mol^{-1}	72.86	83.66	121.12	69.45	69.70	74.55

Supplementary Table 13. Comparison of the terrace and corner fractions before and after terrace peak correction considering electronic structure effect.

	terrace	*terrace corrected*	*corner*	*corner corrected*
AD_0	0.035	0.028	0.126	0.119
AD_1	0.158	0.154	0.108	0.104
AD_5	0.238	0.237	0.072	0.071
NP_0	0.068	0.043	0.176	0.150
NP_1	0.073	0.055	0.145	0.127
NP_5	0.111	0.102	0.127	0.118

Supplementary Table 14. Relative stability of structure with one Ce^{3+} cation with respect to the structure with no Ce^{3+} for the three NP sizes considered here and for a pure CeO_2 surface without nanoparticle, and the charge transferred from Pt to the CeO_2 support, normalized by the number of Pt atoms in the 3D NP.

System	Relative stability (eV)	Charge transferred per Pt atom
Pt0.9	0.43	0.04
Pt1.2	0.37	0.02
Pt1.6	0.47	0.012
CeO_2 (111) without Pt	2.5	

Supplementary Table 15. Parameters extracted from STEM and EEL spectra.

Pt NP size/nm	Area ratio M_5/M_4	Thickness/λ	M_5/M_4 corrected by thickness	M_5/M_4 corrected by Pt-CeO_2 interface contact area
2.54	0.94	0.03	0.028	0.004
1.46	0.96	0.03	0.029	0.014
1.7	0.97	0.03	0.029	0.010
1.5	1.07	0.03	0.032	0.014
2.3	0.91	0.04	0.036	0.007
1.66	1.23	0.03	0.037	0.013
1.7	0.79	0.05	0.039	0.014
1.49	1.00	0.04	0.040	0.018
2.29	1.01	0.04	0.040	0.008
1.67	1.04	0.04	0.042	0.015
2.05	0.86	0.05	0.043	0.010
2.3	1.14	0.04	0.046	0.009
1.16	1.15	0.04	0.046	0.034
1.92	0.94	0.05	0.047	0.013
1.14	1.19	0.04	0.048	0.037
1.77	1.00	0.05	0.050	0.016
1.11	1.13	0.05	0.057	0.046
1.14	1.01	0.06	0.061	0.047
1.42	1.02	0.06	0.061	0.030
1.24	1.05	0.06	0.063	0.041
1.38	1.29	0.05	0.064	0.034
1.32	1.14	0.06	0.068	0.039
1.4	1.00	0.07	0.070	0.036
1.34	0.96	0.08	0.077	0.043

Supplementary Table 16. Previously reported activation energy for WGS reaction over platinum catalysts.

Catalysts	Ea (kJ/mol)	Reference
1%Pt/CeO_2	78.4	Ref[66]
1%Pt/Al_2O_3	68	Ref[37]
1%Pt/Al_2O_3	84	
1.66%Pt/Al_2O_3	81	
1%Pt/CeO_2	75	
1%Pt/CeO_2	91	Ref[67]
1%Pt/CeO_2	90	
Pt/CeO_2/Al_2O_3	76.8	Ref[68]
Pt/CeO_2/Al_2O_3	78.2	
Pt	80	Ref[69]
Pt/CeO_2	80	
2%Pt-1%Re/CeO_2–ZrO_2	71	Ref[70]
2%Pt/Al_2O_3	82	Ref[71]
1%Pt/CeO_2	46	Ref[72]
0.4%Pt/Al_2O_3	39	Ref[73]
1.4%Pt-8.3%CeO_2/Al_2O_3	86	Ref[74]
0.74%Pt/CeO_2	82	Ref[75]
1.15%Pt/VO_x-CeO_2	86	Ref[76]
4.15%Pt/Ce(La)O_x	75	Ref[77]
1wt% Pt_1Na_6/SiO_2(full gas)	65	Ref[59]
1wt% Pt_1Na_6/1000-2h-C_N(full gas)	105	
1 wt% Pt/CZO	85	Ref[78]
2 wt% Pt/CZO	71	
3 wt% Pt/CZO	81	
Pt/CeO_2	54	Ref[79]
Pt/$Ce_{0.8}Zr_{0.2}O_2$	56	
Pt/$Ce_{0.6}Zr_{0.4}O_2$	58	
Pt/$Ce_{0.4}Zr_{0.6}O_2$	64	
Pt/$Ce_{0.2}Zr_{0.8}O_2$	71	
Pt/ZrO_2	83	
Pt(SAs)/CeO_2	84	Ref[63]
Pt(NCs)/CeO_2	79	
Pt(S-NPs)/CeO_2	73	
Pt(M-NPs)/CeO_2	66	
Pt(L-NPs)/CeO_2	66	
0.5Pt/CeO_2	79.9	Ref[80]
0.5Pt/1Ce-10Ti	77.7	
0.5Pt/3Ce-10Ti	74.5	
0.5Pt/5Ce-10Ti	78.6	
0.5Pt/TiO_2	67.5	
0.5Pt/MgO	88.1	Ref[81]
0.5Pt–1$CeO_{2-\delta}$/MgO	70.6	
0.5Pt–2$CeO_{2-\delta}$/MgO	59.4	
0.5Pt–5$CeO_{2-\delta}$/MgO	40.0	
0.5Pt–10$CeO_{2-\delta}$/MgO	34.5	
0.5Pt–20$CeO_{2-\delta}$/MgO	28.8	
0.5Pt/CeO_2	18.4	

Supplementary Table 17. Peak fitting results of XP spectra collected in $CO+H_2O$ and $CO+H_2O+CO_2+H_2$ at 250 °C.

Sample name	*Pt 4f* species proportion				
	Bulk	Terrace	Edge	AD	Corner
NP_0-$CO+H_2O$	0.082	0.068	0.024	0.650	0.176
NP_0-$CO+H_2O+CO_2+H_2$	0.079	0.065	0.023	0.661	0.172
NP_5-$CO+H_2O$	0.130	0.111	0.079	0.552	0.127
NP_5-$CO+H_2O+CO_2+H_2$	0.140	0.119	0.084	0.527	0.130

Supplementary references

1. Tao F*, et al.* Break-Up of Stepped Platinum Catalyst Surfaces by High CO Coverage. *Science* **327**, 850-853 (2010).
2. James G, Witten D, Hastie T, Tibshirani R. Introduction. In: *An Introduction to Statistical Learning: with Applications in R* (eds James G, Witten D, Hastie T, Tibshirani R). Springer New York (2013).
3. Despotovic M, Nedic V, Despotovic D, Cvetanovic S. Evaluation of empirical models for predicting monthly mean horizontal diffuse solar radiation. *Renewable and Sustainable Energy Reviews* **56**, 246-260 (2016).
4. Li M-F, Tang X-P, Wu W, Liu H-B. General models for estimating daily global solar radiation for different solar radiation zones in mainland China. *Energy Convers Manage* **70**, 139-148 (2013).
5. Peralta M. *Propagation of errors: how to mathematically predict measurement errors to first and second order*. Selbstverl. (2013).
6. Pereira-Hernández XI*, et al.* Tuning Pt-CeO 2 interactions by high-temperature vapor-phase synthesis for improved reducibility of lattice oxygen. *Nat Commun* **10**, 1358 (2019).
7. Bera P*, et al.* Promoting effect of CeO2 in combustion synthesized Pt/CeO2 catalyst for CO oxidation. *J Phys Chem B* **107**, 6122-6130 (2003).
8. Dauscher A, Hilaire L, Le Normand F, Müller W, Maire G, Vasquez A. Characterization by XPS and XAS of supported Pt/TiO2□ CeO2 catalysts. *Surf Interface Anal* **16**, 341-346 (1990).
9. Arble C, Jia M, Newberg JT. Lab-based ambient pressure X-ray photoelectron spectroscopy from past to present. *Surf Sci Rep* **73**, 37-57 (2018).
10. Winterbottom WL. Equilibrium shape of a small particle in contact with a foreign substrate. *Acta Metall* **15**, 303-310 (1967).
11. Vincent JL, Crozier PA. Atomic level fluxional behavior and activity of CeO2-supported Pt catalysts for CO oxidation. *Nat Commun* **12**, 5789 (2021).
12. McCrum IT, Hickner MA, Janik MJ. First-principles calculation of Pt surface energies in an electrochemical environment: thermodynamic driving forces for surface faceting and nanoparticle reconstruction. *Langmuir* **33**, 7043-7052 (2017).
13. Ding R*, et al.* Size-dependent shape distributions of platinum nanoparticles. *Nanoscale Advances* **4**, 3978-3986 (2022).
14. Lai KC, Campbell CT, Evans JW. Size-dependent diffusion of supported metal nanoclusters: mean-field-type treatments and beyond for faceted clusters. *Nanoscale Horizons* **8**, 1556-1567 (2023).
15. Barnard A, Konishi H, Xu H. Morphology mapping of platinum catalysts over the entire nanoscale. *Catalysis Science & Technology* **1**, 1440-1448 (2011).
16. Ding R*, et al.* Size-dependent shape distributions of platinum nanoparticles. *Nanoscale advances* **4**, 3978-3986 (2022).
17. Crozier PA*, et al.* Visualizing nanoparticle surface dynamics and instabilities enabled by deep denoising. *Science* **387**, 949-954 (2025).
18. Li Y*, et al.* Dynamic structure of active sites in ceria-supported Pt catalysts for the water gas shift reaction. *Nat Commun* **12**, 914 (2021).
19. Li G*, et al.* Tracking the dynamics of catalytic Pt/CeO2 active sites during water-gas-shift reaction. *Communications Materials* **5**, 133 (2024).
20. Avanesian T, Dai S, Kale MJ, Graham GW, Pan X, Christopher P. Quantitative and Atomic-Scale View of CO-Induced Pt Nanoparticle Surface

Reconstruction at Saturation Coverage via DFT Calculations Coupled with in Situ TEM and IR. *J Am Chem Soc* **139**, 4551-4558 (2017).
21. Wulff G. Velocity of growth and dissolution of crystal faces. *Z Kristallogr* **34**, 449-530 (1901).
22. Henry CR. Morphology of supported nanoparticles. *Prog Surf Sci* **80**, 92-116 (2005).
23. Wang X, Van Bokhoven JA, Palagin D. Atomically dispersed platinum on low index and stepped ceria surfaces: phase diagrams and stability analysis. *Phys Chem Chem Phys* **22**, 28-38 (2020).
24. Torrente-Murciano L. The importance of particle-support interaction on particle size determination by gas chemisorption. *J Nanopart Res* **18**, 87 (2016).
25. Liu J. Advanced Electron Microscopy of Metal–Support Interactions in Supported Metal Catalysts. *ChemCatChem* **3**, 934-948 (2011).
26. Aranifard S, Ammal SC, Heyden A. On the importance of metal–oxide interface sites for the water–gas shift reaction over Pt/CeO_2 catalysts. *J Catal* **309**, 314-324 (2014).
27. Aranifard S, Ammal SC, Heyden A. On the Importance of the Associative Carboxyl Mechanism for the Water-Gas Shift Reaction at Pt/CeO_2 Interface Sites. *J Phys Chem C* **118**, 6314-6323 (2014).
28. Bruix A*, et al.* A New Type of Strong Metal–Support Interaction and the Production of H_2 through the Transformation of Water on Pt/CeO_2(111) and Pt/CeO_x/TiO_2(110) Catalysts. *J Am Chem Soc* **134**, 8968-8974 (2012).
29. Lykhach Y*, et al.* Counting electrons on supported nanoparticles. *Nat Mater* **15**, 284-288 (2016).
30. Kalamaras CM, Americanou S, Efstathiou AM. “Redox” vs “associative formate with –OH group regeneration” WGS reaction mechanism on Pt/CeO_2: Effect of platinum particle size. *J Catal* **279**, 287-300 (2011).
31. Meunier FC, Tibiletti D, Goguet A, Shekhtman S, Hardacre C, Burch R. On the complexity of the water-gas shift reaction mechanism over a Pt/CeO_2 catalyst: Effect of the temperature on the reactivity of formate surface species studied by operando DRIFT during isotopic transient at chemical steady-state. *Catal Today* **126**, 143-147 (2007).
32. Kalamaras CM, Petallidou KC, Efstathiou AM. The effect of La^{3+}-doping of CeO_2 support on the water-gas shift reaction mechanism and kinetics over Pt/$Ce_{1-x}La_xO_{2-\delta}$. *Applied Catalysis B: Environmental* **136-137**, 225-238 (2013).
33. Kalamaras CM, Gonzalez ID, Navarro RM, Fierro JLG, Efstathiou AM. Effects of Reaction Temperature and Support Composition on the Mechanism of Water–Gas Shift Reaction over Supported-Pt Catalysts. *J Phys Chem C* **115**, 11595-11610 (2011).
34. Jacobs G, Khalid S, Patterson PM, Sparks DE, Davis BH. Water-gas shift catalysis: kinetic isotope effect identifies surface formates in rate limiting step for Pt/ceria catalysts. *Applied Catalysis A: General* **268**, 255-266 (2004).
35. Li X. Structure-Performance Relationship of Pt/CeO2 Catalysts for the Water-Gas Shift Reaction.). ETH Zurich (2023).
36. Grabow LC, Gokhale AA, Evans ST, Dumesic JA, Mavrikakis M. Mechanism of the Water Gas Shift Reaction on Pt: First Principles, Experiments, and Microkinetic Modeling. *J Phys Chem C* **112**, 4608-4617 (2008).

37. Phatak AA, *et al.* Kinetics of the water-gas shift reaction on Pt catalysts supported on alumina and ceria. *Catal Today* **123**, 224-234 (2007).
38. Lu J-B, Jiang X-L, Hu H-S, Li J. Norm-Conserving 4f-in-Core Pseudopotentials and Basis Sets Optimized for Trivalent Lanthanides (Ln = Ce–Lu). *Journal of Chemical Theory and Computation* **19**, 82-96 (2023).
39. Kozlov SM, Neyman KM. Effects of electron transfer in model catalysts composed of Pt nanoparticles on CeO_2(111) surface. *J Catal* **344**, 507-514 (2016).
40. Lee J, *et al.* How Pt Influences H2 Reactions on High Surface-Area Pt/CeO2 Powder Catalyst Surfaces. *JACS Au* **3**, 2299-2313 (2023).
41. Jenkinson K, *et al.* Direct Operando Visualization of Metal Support Interactions Induced by Hydrogen Spillover During CO2 Hydrogenation. *Advanced Materials* **35**, 2306447 (2023).
42. Garvie LAJ, Buseck PR. Determination of Ce4+/Ce3+ in electron-beam-damaged CeO2 by electron energy-loss spectroscopy. *Journal of Physics and Chemistry of Solids* **60**, 1943-1947 (1999).
43. Abruña HD, Gao J, Holtz ME, Muller DA, Yu Y. In Situ Electron Energy-Loss Spectroscopy in Liquids. *Microscopy and Microanalysis* **19**, 1027-1035 (2013).
44. Oh-ishi K, Ohsuna T. Inelastic mean free path measurement by STEM-EELS technique using needle-shaped specimen. *Ultramicroscopy* **212**, 112955 (2020).
45. Mann HB, Whitney DR. On a test of whether one of two random variables is stochastically larger than the other. *The annals of mathematical statistics*, 50-60 (1947).
46. Welch B. THE GENERALIZATION OF'STUDENT'S'PROBLEM WHEN SEVERAL DIFFERENT POPULATION. (1947).
47. Pazmino JH, *et al.* Metallic Pt as active sites for the water-gas shift reaction on alkali-promoted supported catalysts. *J Catal* **286**, 279-286 (2012).
48. Ghosalya MK, Li X, Beck A, van Bokhoven JA, Artiglia L. Size of Ceria Particles Influences Surface Hydroxylation and Hydroxyl Stability. *J Phys Chem C* **125**, 9303-9309 (2021).
49. Jung M, *et al.* Surface study of Pt-3d transition metal alloys, Pt3M (M = Ti, V), under CO oxidation reaction with ambient pressure x-ray photoelectron spectroscopy. *Journal of Vacuum Science & Technology A* **39**, (2021).
50. Yu Y, *et al.* Chemical states of surface oxygen during CO oxidation on Pt(1 1 0) surface revealed by ambient pressure XPS. *J Phys: Condens Matter* **29**, 464001 (2017).
51. Peng Y, *et al.* Alkali Metal Poisoning of a CeO_2–WO_3 Catalyst Used in the Selective Catalytic Reduction of NOx with NH_3: an Experimental and Theoretical Study. *Environmental Science & Technology* **46**, 2864-2869 (2012).
52. Ang ML, *et al.* Highly Active Ni/xNa/CeO_2 Catalyst for the Water–Gas Shift Reaction: Effect of Sodium on Methane Suppression. *ACS Catal* **4**, 3237-3248 (2014).
53. Li Y, Wei Z, Sun J, Gao F, Peden CHF, Wang Y. Effect of Sodium on the Catalytic Properties of VO_x/CeO_2 Catalysts for Oxidative Dehydrogenation of Methanol. *J Phys Chem C* **117**, 5722-5729 (2013).

54. Pashalidis I, Theocharis CR. Investigations on the Surface Properties of Pure and Alkali or Alkaline Earth Metal Doped Ceria. In: *Studies in Surface Science and Catalysis* (eds Unger KK, Kreysa G, Baselt JP). Elsevier (2000).
55. Kusche M, Bustillo K, Agel F, Wasserscheid P. Highly Effective Pt‐Based Water‐Gas Shift Catalysts by Surface Modification with Alkali Hydroxide Salts. *ChemCatChem* **7**, 766-775 (2015).
56. Ratnasamy C, Wagner JP. Water Gas Shift Catalysis. *Catalysis Reviews* **51**, 325-440 (2009).
57. Zhai YP*, et al.* Alkali-Stabilized Pt-OH_x Species Catalyze Low-Temperature Water-Gas Shift Reactions. *Science* **329**, 1633-1636 (2010).
58. Yang M*, et al.* Catalytically active Au-O$(OH)_{(x)}$-species stabilized by alkali ions on zeolites and mesoporous oxides. *Science* **346**, 1498-1501 (2014).
59. Zugic B, Zhang S, Bell DC, Tao F, Flytzani-Stephanopoulos M. Probing the Low-Temperature Water–Gas Shift Activity of Alkali-Promoted Platinum Catalysts Stabilized on Carbon Supports. *J Am Chem Soc* **136**, 3238-3245 (2014).
60. Petallidou KC, Polychronopoulou K, Boghosian S, Garcia-Rodriguez S, Efstathiou AM. Water–Gas Shift Reaction on Pt/$Ce_{1-x}TixO_{2-\delta}$: The Effect of Ce/Ti Ratio. *J Phys Chem C* **117**, 25467-25477 (2013).
61. Resasco J*, et al.* Uniformity Is Key in Defining Structure–Function Relationships for Atomically Dispersed Metal Catalysts: The Case of Pt/CeO_2. *J Am Chem Soc* **142**, 169-184 (2020).
62. Qiao B*, et al.* Single-atom catalysis of CO oxidation using Pt_1/FeOx. *Nat Chem* **3**, 634-641 (2011).
63. Yuan K*, et al.* Size effect-tuned water gas shift reaction activity and pathway on ceria supported platinum catalysts. *J Catal* **394**, 121-130 (2021).
64. Chen MS, Goodman DW. The Structure of Catalytically Active Gold on Titania. *Science* **306**, 252-255 (2004).
65. Pedregosa F*, et al.* Scikit-learn: Machine learning in Python. *the Journal of machine Learning research* **12**, 2825-2830 (2011).
66. Park YM, Son M, Park M-J, Bae JW. Effects of Pt precursors on Pt/CeO_2 to water-gas shift (WGS) reaction activity with Langmuir-Hinshelwood model-based kinetics. *Int J Hydrogen Energy* **45**, 26953-26966 (2020).
67. Thinon O, Rachedi K, Diehl F, Avenier P, Schuurman Y. Kinetics and mechanism of the water–gas shift reaction over platinum supported catalysts. *Top Catal* **52**, 1940-1945 (2009).
68. Germani G, Schuurman Y. Water‐gas shift reaction kinetics over μ‐structured Pt/CeO_2/Al_2O_3 catalysts. *AlChE J* **52**, 1806-1813 (2006).
69. Wheeler C, Jhalani A, Klein E, Tummala S, Schmidt L. The water–gas-shift reaction at short contact times. *J Catal* **223**, 191-199 (2004).
70. Radhakrishnan R, Willigan R, Dardas Z, Vanderspurt T. Water gas shift activity and kinetics of Pt/Re catalysts supported on ceria-zirconia oxides. *Applied catalysis B: environmental* **66**, 23-28 (2006).
71. Grenoble D, Estadt M, Ollis D. The chemistry and catalysis of the water gas shift reaction: 1. The kinetics over supported metal catalysts. *J Catal* **67**, 90-102 (1981).
72. Bunluesin T, Gorte R, Graham G. Studies of the water-gas-shift reaction on ceria-supported Pt, Pd, and Rh: implications for oxygen-storage properties. *Applied Catalysis B: Environmental* **15**, 107-114 (1998).

73. Lam C, Stacey M, Trimm D. The combustion of methane on platinum-alumina fibre catalysts—III. The kinetics of the water gas shift reaction. *Chem Eng Sci* **36**, 224-226 (1981).
74. Germani G, Alphonse P, Courty M, Schuurman Y, Mirodatos C. Platinum/ceria/alumina catalysts on microstructures for carbon monoxide conversion. *Catal Today* **110**, 114-120 (2005).
75. Miao D, Goldbach A, Xu H. Platinum/apatite water-gas shift catalysts. *ACS Catal* **6**, 775-783 (2016).
76. de Farias AMD, Bargiela P, Maria da Graça CR, Fraga MA. Vanadium-promoted Pt/CeO_2 catalyst for water–gas shift reaction. *J Catal* **260**, 93-102 (2008).
77. Fu Q, Saltsburg H, Flytzani-Stephanopoulos M. Active nonmetallic Au and Pt species on ceria-based water-gas shift catalysts. *Science* **301**, 935-938 (2003).
78. Lim S, Bae J, Kim K. Study of activity and effectiveness factor of noble metal catalysts for water–gas shift reaction. *Int J Hydrogen Energy* **34**, 870-876 (2009).
79. Jeong D-W, Potdar HS, Shim J-O, Jang W-J, Roh H-S. H_2 production from a single stage water–gas shift reaction over Pt/CeO_2, Pt/ZrO_2, and Pt/$Ce_{(1-x)}Zr_{(x)}O_2$ catalysts. *Int J Hydrogen Energy* **38**, 4502-4507 (2013).
80. Lai X-M, Xiao Q, Ma C, Wang W-W, Jia C-J. Heterostructured Ceria–Titania-Supported Platinum Catalysts for the Water Gas Shift Reaction. *ACS Appl Mater Interfaces* **14**, 8575-8586 (2022).
81. Wang Y*, et al.* Complete CO Oxidation by O_2 and H_2O over Pt–$CeO_{2-\delta}$/MgO Following Langmuir–Hinshelwood and Mars–van Krevelen Mechanisms, Respectively. *ACS Catal* **11**, 11820-11830 (2021).
