# Supplementary material for: Quantifying electronic and geometric effects on the activity of platinum catalysts for water-gas shift
Source: Nat Commun. 2025 Jul 18;16:6641. doi: 10.1038/s41467-025-61895-8 (PMC12274351; doi:10.1038/s41467-025-61895-8)
Supplement: Supplementary file 2 — Description of Additional Supplementary Files [file 41467_2025_61895_MOESM2_ESM.pdf]

Description of Additional Supplementary Files

File Name: Supplementary Movie 1
Description: Dynamics of NP_0 catalyst in 0.2 mbar CO_2 + 0.2 mbar H_2 at 250 °C. The labeled time stamp is in mm:ss.

File Name: Supplementary Movie 2
Description: Dynamics of NP_0 catalyst in 0.25 bar CO_2 + 0.75 bar H_2 at 300 °C. The labeled time stamp is in mm:ss.

File Name: Supplementary Movie 3
Description: Dynamics of the model Pt/CeO_2 catalyst at 300 °C in 0.25 bar CO_2 + 0.75 bar H_2. The labeled time stamp is in mm:ss.
